# An* Myh11* single lysine deletion causes aortic dissection by reducing aortic structural integrity and contractility

**DOI:** 10.1038/s41598-022-12418-8

**Published:** 2022-05-25

**Authors:** Keita Negishi, Kenichi Aizawa, Takayuki Shindo, Toru Suzuki, Takayuki Sakurai, Yuichiro Saito, Takuya Miyakawa, Masaru Tanokura, Yosky Kataoka, Mitsuyo Maeda, Shota Tomida, Hiroyuki Morita, Norifumi Takeda, Issei Komuro, Kazuomi Kario, Ryozo Nagai, Yasushi Imai

**Affiliations:** 1 Division of Clinical Pharmacology, Department of Pharmacology, Jichi Medical University, 3311-1 Yakushiji, Shimotsuke, Tochigi, 329-0498 Japan; 2 Division of Cardiovascular Medicine, Department of Medicine, Jichi Medical University, Tochigi, Japan; 3 Department of Cardiovascular Research, Shinshu University Graduate School of Medicine, Nagano, Japan; 4 Department of Cardiovascular Sciences, University of Leicester Cardiovascular Research Centre, Glenfield Hospital, Leicester, UK; 5 System Integration Center, Gunma University Hospital, Gunma, Japan; 6 Department of Applied Biological Chemistry, Graduate School of Agricultural and Life Sciences, The University of Tokyo, Tokyo, Japan; 7 RIKEN Center for Biosystems Dynamics Research, Kobe, Japan; 8 RIKEN-JEOL Collaboration Center, Kobe, Japan; 9 Department of Cardiovascular Medicine, Graduate School of Medicine, The University of Tokyo, Tokyo, Japan; 10 Jichi Medical University, 3311-1 Yakushiji, Shimotsuke, Tochigi, 329-0498 Japan

**Keywords:** Aortic diseases, Cardiovascular genetics, Experimental models of disease

## Abstract

Pathogenic variants in myosin heavy chain (*Myh11*) cause familial thoracic aortic aneurysms and dissections (FTAAD). However, the underlying pathological mechanisms remain unclear because of a lack of animal models. In this study, we established a mouse model with *Myh11* K1256del, the pathogenic variant we found previously in two FTAAD families. The *Myh11*^∆K/∆K^ aorta showed increased wall thickness and ultrastructural abnormalities, including weakened cell adhesion. Notably, the *Myh11*^∆K/+^ mice developed aortic dissections and intramural haematomas when stimulated with angiotensin II. Mechanistically, integrin subunit alpha2 (*Itga2*) was downregulated in the *Myh11*^∆K/∆K^ aortas, and the smooth muscle cell lineage cells that differentiated from *Myh11*^∆K/∆K^ induced pluripotent stem cells. The contractility of the *Myh11*^∆K/∆K^ aortas in response to phenylephrine was also reduced. These results imply that the suboptimal cell adhesion indicated by *Itga2* downregulation causes a defect in the contraction of the aorta. Consequently, the defective contraction may increase the haemodynamic stress underlying the aortic dissections.

## Introduction

At least 20% of thoracic aortic disease patients without syndromic features have an affected first-degree relative, known as familial thoracic aortic aneurysms and dissections (FTAAD)^1–3^. Four pathogenic genes associated with smooth muscle contractile function (*ACTA2*, *MYH11*, *MYLK* and *PRKG1*) have been identified^4–7^. Although FTAAD is a non-syndromic thoracic aortic disease, patients with pathogenic variants in those genes present aortic phenotypes similar to those of syndromic thoracic aortic diseases^8^. *MYH11* encodes smooth muscle specific myosin heavy chain (SM-MHC), and the pathogenic variants in *MYH11* were reported in 2% of families with FTAAD/patent ductus arteriosus (PDA)^9^. Pathogenic variants are found mainly in the C-terminal coiled-coil region of SM-MHC and are predicted to affect the polymerisation of thick filaments^4^. Genetic alteration in *Myh11*, even the rare variant or copy-number variant identified in large populations, disrupts the cytoskeletal and contractile function of smooth muscle cells (SMCs) and increases intracellular stress in vitro, leading to remodelling in thoracic aortic disease^10–12^. However, the pathogenesis of how the *Myh11* pathogenic variant leads to aortic dissection remains unclear because of a lack of appropriate animal models.

Recently, we reported a deletion variant (K1256del) in *MYH11*, which disrupts the four-lysine-alignment of K1253–K1256 in the region of light-meromyosin, and it was completely conserved across species in two Japanese FTAAD pedigrees which were independent of each other^13^. In this study, we developed a murine model of this *Myh11* K1256del pathogenic variant using the CRISPR-Cas9 system to evaluate the pathogenesis of FTAAD originating from the *Myh11* pathogenic variant.

## Results

### Generation of *Myh11* 1256 K-deficient mice

**Figure 1:**
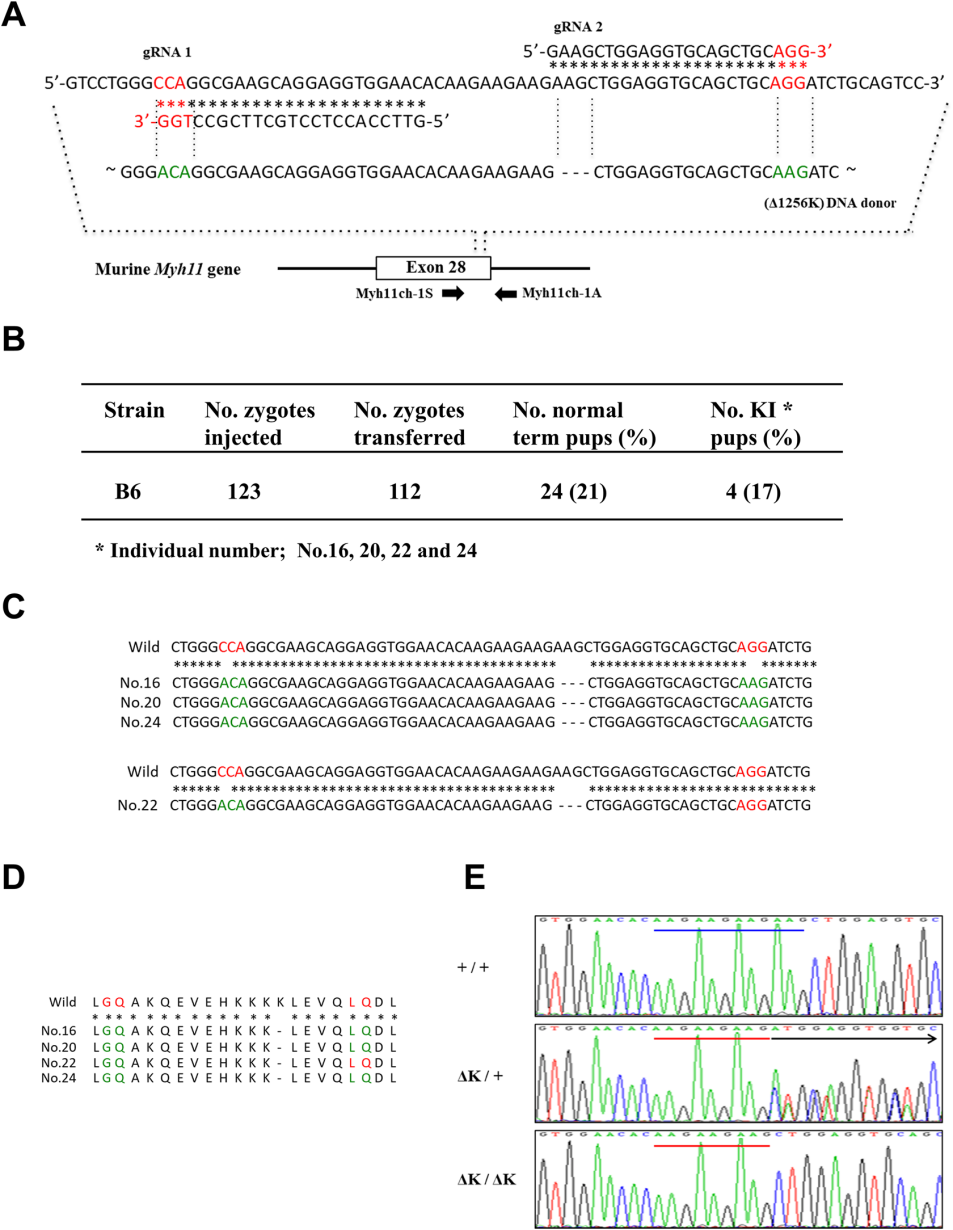
CRISPR/Cas9-based generation of *Myh11* (Δ1256K) mice.** (A)** Sequence of exon 28 of murine *Myh11* to which both gRNA1 and 2 bind and an *Myh11* (Δ1256K) DNA donor targeting site. The PAM and modified PAM sequence site in the *Myh11* (Δ1256K) DNA donor fragment are indicated by red and green, respectively. Locations of PCR primers are indicated by arrows. **(B)** Table summarising the number (no.) of zygotes injected, no. zygotes transferred and no. zygotes developed to full term is shown. The no. of pups showing knock-in is also shown. **(C)** Sequences around the *Myh11* target site in wild-type (WT) and knock-in samples (nos. 16, 20, 22 and 24). Upper panel shows sequences with the deletion of AAG alone and those in which both PAM sequences (red) were changed to modified PAM sequences (green) in the samples numbered 16, 20 and 24. Lower panel shows the sequences showing the deletion of AAG alone and those in which only a PAM sequence recognised by gRNA1 (red) was changed to modified PAM sequences (green) recognised by gRNA1 in the sample numbered 22. **(D)** Amino acid sequences around the *Myh11* target site of WT and knock-in samples (nos. 16, 20, 22 and 24). Note that the sequences of all knock-in pups are the same as those of the WT pups except for the absence of lysine (K) in knock-in pups. Red shows amino acids corresponding to WT PAM sequences. Green shows amino acids corresponding to modified PAM sequences. The no. 22 line with WT PAM sequences recognised by gRNA2 was selected for further analysis because its alleles are close to the WT *Myh11* allele. The no. 22 line was backcrossed to B6 background through passing 2 generations. **(E)** *Myh11* (Δ1256K) genotyping of the no. 22 line by direct sequencing of PCR products corresponding to the *Myh11* target site. The blue line shows AAG × 4, the red line shows AAG × 3 and the arrow shows the start of error ideograms.

To investigate the pathological effects of the deletion variant of *Myh11* 1256 K, we attempted to introduce this variant into B6 mice using the CRISPR-Cas9 system, but this gene manipulation resulted in embryonic lethality. The cause of embryonic lethality was not investigated further. To reduce the off-target effects of the CRISPR-Cas9 system, we used Cas9 (D10A) mRNA to generate four founder mice (Fig. 1B): three heterozygotes (one male and two females) and one male homozygote (Fig. 1C, D). The heterozygous breeding pairs were used to generate homozygotes, but they failed to foster their pups. The cause of neglect was not studied and remains unknown. The founder homozygote suddenly died at four weeks old (of uncertain cause but not aortic disease). We next collected sperm from the dead homozygote, which was used for in vitro fertilisation-embryo transfer with B6 females. As a result, we generated five heterozygotes of the next generation and backcrossed them more than three times. Subsequently, wild-type (WT), heterozygous (*Myh11*^∆K/+^) and homozygous (*Myh11*^∆K/∆K^) mice obtained by line crossing were used for analysis. The genotypes of all mice were determined by a DNA sequence analysis of the genetically modified site (Fig. 1E).

*Myh11*^∆K/+^ females became pregnant normally, and their mutant pups showed Mendelian ratios; however, perinatal issues, such as infanticide and abandonment, occasionally occurred. In *Myh11*^∆K/∆K^ mothers, stillbirth was more likely to occur than in WT (Supplementary Fig. 1A), and even homozygous mothers often died during delivery because the delivery time was prolonged for more than 24 h (Supplementary Fig. 1B). Because we generated a relatively small number of *Myh11*^∆K/∆K^ males by natural mating, we used in vitro fertilisation-embryo transfer to generate homozygotes for our experiments, and the surrogate mothers for the embryo transfer carried and raised their pups normally. After weaning, the *Myh11*^∆K/∆K^ pups grew normally and lived for more than 18 months without aortic dissection (Supplementary Fig. 1C).

### *Myh11*^∆K/∆K^ aorta showed thickened media and adventitia

**Figure 2:**
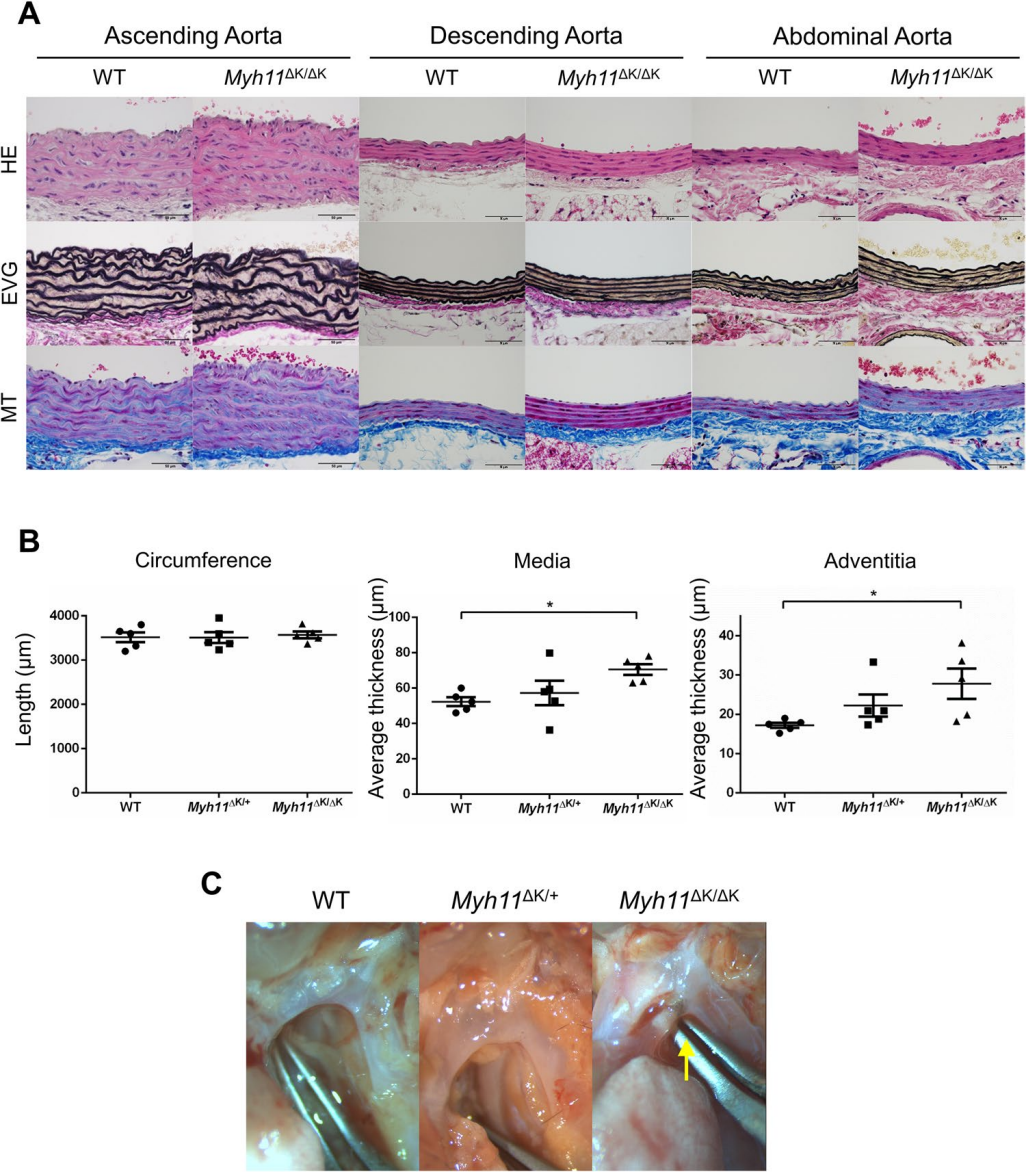
*Myh11*^∆K/∆K^ ascending aortas showed phenotypes of medial degeneration.** (A)** Representative architecture of aortic wall (ascending, descending and abdominal aorta) from 12-week-old WT and *Myh11*^∆K/∆K^ mice. Cross-sections were stained with haematoxylin and eosin (HE), Elastica van Gieson (EVG, for elastin) and Masson trichome (MT, for collagen; scale bars = 50 µm, × 400). *Myh11*^∆K/∆K^ aortas showed partial tears of elastic fibres and an increased thickness of media and adventitia. **(B)** Morphometric parameters of WT, *Myh11*^∆K/+^ and *Myh11*^∆K/∆K^ ascending thoracic aortas. Circumference length did not differ between the WT, *Myh11*^∆K/+^ and *Myh11*^∆K/∆K^ aortas (left, n = 5), while the media thickness (middle, n = 5) and adventitia thickness (right, n = 5) of the *Myh11*^∆K/∆K^ aortas were significantly increased compared to those of the WT aortas. ** p < 0.01, one-way ANOVA with Tukey post-hoc test. **(C)** Gross appearance of a shunt vessel between the distal arch and pulmonary artery, considered as patent ductus arteriosus (PDA), in 12-week-old *Myh11*^∆K/∆K^ mice.

To evaluate the structural features of *Myh11*^∆K/∆K^ aortas, we excised the aortas (ascending, descending and abdominal) from WT, *Myh11*^∆K/+^ and *Myh11*^∆K/∆K^ mice at 12 weeks of age (n = 5) for histological observation. The *Myh11*^∆K/∆K^ aortas showed increased medial thickness (WT vs *Myh11*^∆K/∆K^, p < 0.05) and thickened adventitia (p < 0.05) but no significantly expanded aortic lumen (p = 0.30). Conversely, the *Myh11*^∆K/+^ aortas did not show any statistically significant increase in the thickness of the media or adventitia compared to the WT aortas (Fig. 2A, B and Supplementary Fig. 2). None of the aortic regions showed a higher rate of dissection compared to the other regions (Supplementary Table 1).

In all *Myh11*^∆K/∆K^ mice dissected, a shunt blood vessel connecting the distal aortic arch and pulmonary artery was observed (Fig. 2C) and resembled PDA, which is frequently associated with FTAAD. Furthermore, an enlarged bladder, swollen kidneys indicating hydronephrosis and hypoplastic uteri were observed in the *Myh11*^∆K/∆K^ mice (Supplementary Fig. 3A). Non-uniform staining, such as punch-out and gaps between the smooth muscle layers, was commonly observed in *Myh11*^∆K/∆K^ bladders (Supplementary Fig. 3B). Quantifying the thickness of the smooth muscle layers (myometria), we observed that the myometrium of *Myh11*^∆K/∆K^ uterus were thinner than those of the WT (Supplementary Fig. 3C and D).

### Cell adhesion of SMCs and composition of ECM are decreased in *Myh11* mutant aortas

**Figure 3:**
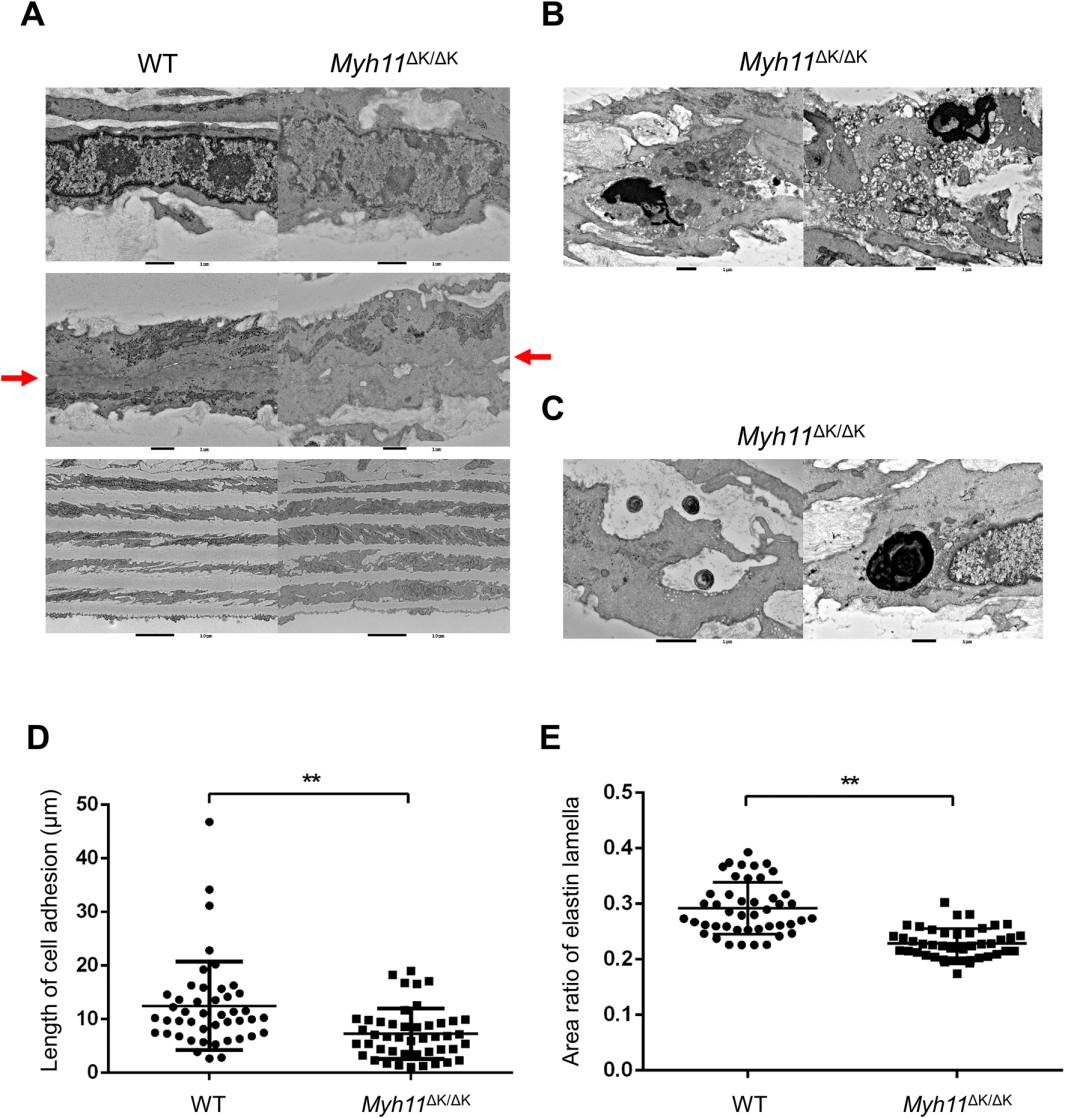
Large-scale electron microscopy indicated increased intracellular stress, decreased elastin lamella and attenuated cell adhesion in *Myh11*^∆K/∆K^ aortas.** (A)** Electron microscopy images of WT and *Myh11*^∆K/∆K+^ descending aortas. *Myh11*^∆K/∆K^ nuclei included thinner nuclear envelope and many granular components (upper row, scale bars = 1 µm, × 10,000). The adhesive faces between SMCs in *Myh11*^∆K/∆K+^ aortas were shorter than those in WT (middle row, scale bars = 1 µm, × 12,000). The size of the SMCs in the *Myh11*^∆K/∆K+^ aortas were larger than those in WT (bottom row, scale bars = 10 µm, × 2,000). **(B)** Debris was occasionally found in the *Myh11*^∆K/∆K^ aortas (scale bars = 1 µm, × 10,000). **(C)** Myelin figures were present inside or outside *Myh11*^∆K/∆K^ SMCs [scale bars = 1 µm, × 12,000 (left), × 20,000 (right)]. **(D)** The adhesive length of the SMCs in the *Myh11*^∆K/∆K^ aorta was significantly shorter than that in WT [n = 45 (15 cells per an aortic sample), WT 12.50 ± 8.16 µm/cell vs *Myh11*^∆K/∆K^ 7.28 ± 4.66 µm/cell, p < 0.01). **(E)** The area ratio of the elastic lamellae in the 50-µm section was decreased in *MYH11*^∆K/∆K^ aortas compared to that in WT [n = 45 (15 of 50-µm sections per an aortic sample), WT 0.29 ± 0.04 vs *Myh11*^∆K/∆K^ 0.23 ± 0.02, p < 0.01]. ** p < 0.01, Mann–Whitney U test.

To analyse the aortic microstructures, we examined the descending aortas of WT and *Myh11*^∆K/∆K^ mice at 18 weeks of age by electron microscopy assuming that each aortic region was similarly predisposed to dissection since occurrence of dissection was not biased towards a specific region (Supplementary Table 1). The results revealed a thinner nuclear envelope and numerous granular components in the mutant nucleus (Fig. 3A, upper row), which appeared in the nuclear morphology as prevalent euchromatin and less heterochromatin, indicating active transcriptional activities. These nuclear features suggested a latent shift from the contractile phenotype to the synthetic phenotype in a part of mutant SMCs. *Myh11*^∆K/∆K^ SMCs also showed an attenuation of cell adhesion to other adjacent cells and were larger than WT SMCs (Fig. 3A, middle and bottom rows). Furthermore, we detected some of features often observed in dead cells in *Myh11*^∆K/∆K^ SMCs. Increased organelles were often observed in *Myh11*^∆K/∆K^ SMCs, and debris was occasionally detected (Fig. 3B). We also found concentrically layered osmiophilic material, known as myelin figures, inside or outside the mutant SMCs, which were quite rare in WT (Fig. 3C).

To evaluate the structural integrity of the aortas, we further analysed the cell adhesion and elastin lamella by using comprehensive images of cross-sectional aortas, which were obtained from large-scale electron microscopic images. We measured the length of the intercellular adhesions of longitudinally sectioned SMCs (Supplementary Fig. 4A; a detailed protocol is provided in the supplementary material online), and the intercellular adhesions were significantly shortened in *Myh11*^∆K/∆K^ aortas (WT vs *Myh11*^∆K/∆K^, p < 0.01, Fig. 3D). The area of elastic lamellas, identified as an area of plain texture (Supplementary Fig. 4B; a detailed protocol is provided in the supplementary material online) was also decreased in *Myh11*^∆K/∆K^ aortas (p < 0.01, Fig. 3E).

### Contractile function was attenuated in *Myh11*^∆K/∆K^ SMCs

**Figure 4:**
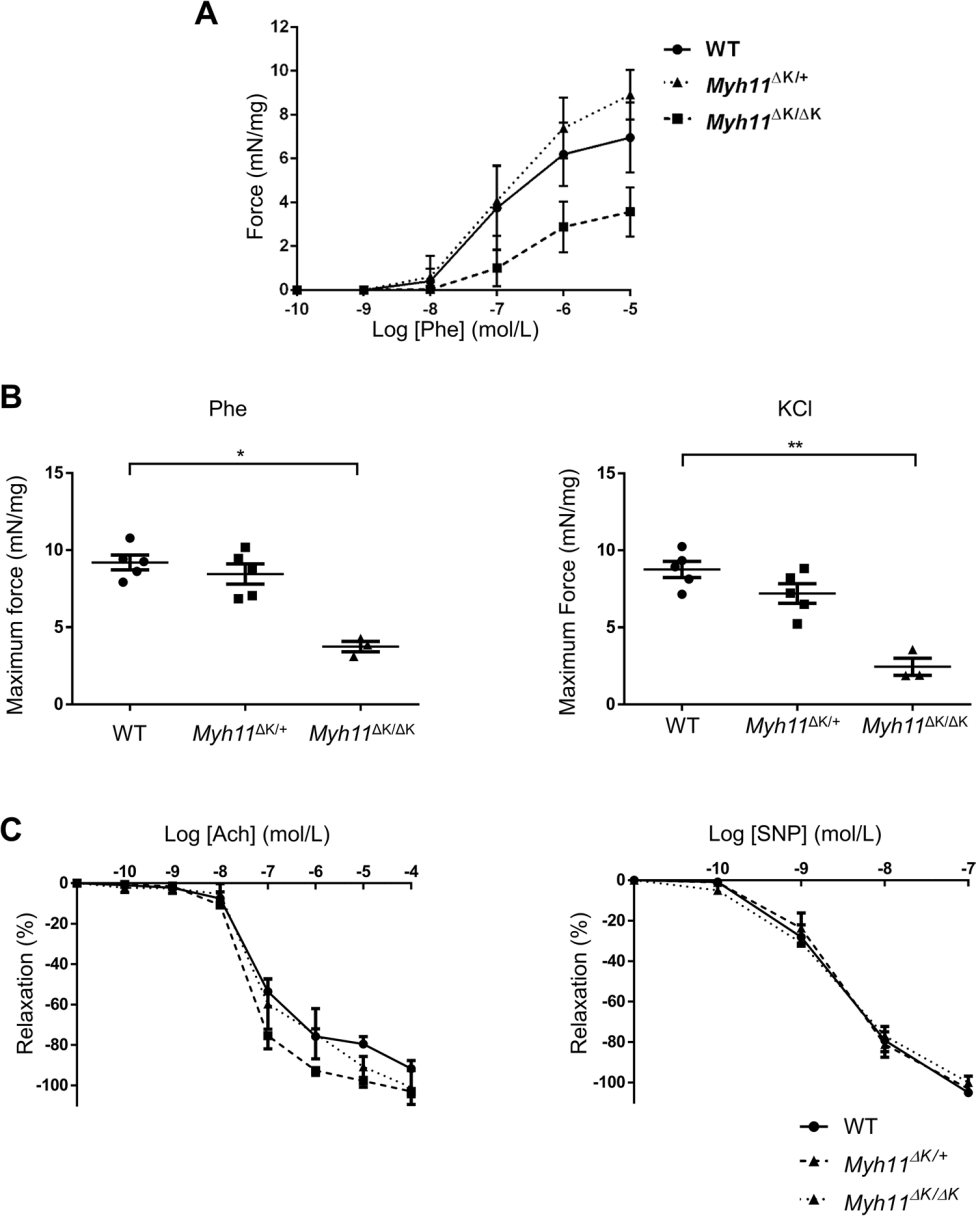
*Myh11* K1256del attenuated contractile function in *Myh11*^∆K/∆K^ SMCs.** (A)** Effect of *Myh11* K1256del on the dose-dependent contraction of aortas induced by phenylephrine (Phe). Each point represents the mean ± SEM. **(B)** Scatter dot plot showing the means ± SD of maximum force that the WT, *Myh11*^∆K/+^ and *Myh11*^∆K/∆K^ aortas produced after phenylephrine (Phe) or KCl treatment (WT and *Myh11*^∆K/+^: n = 5; *Myh11*^∆K/∆K^: n = 3). * p < 0.05, Kruskal–Wallis test followed by Dunn’s multiple comparisons test. **(C)** Rate of relaxation of descending aortas in response to acetylcholine (ACh) and sodium nitroprusside (SNP) after the contractile response to 10^–5^ mol/L Phe (WT and *Myh11*^∆K/+^: n = 5; *Myh11*^∆K/∆K^: n = 3). There is no significant difference in the endothelium-dependent or endothelium-independent vasodilation between the WT and *Myh11*^∆K/+^ aortas.

Considering these morphological features in the *Myh11*^∆K/∆K^ aortas, we hypothesised that *Myh11* K1256del negatively affects SMC function. To evaluate SMC contractility in the aortas, we measured the isometric force of aortic rings from WT, *Myh11*^∆K/+^ and *Myh11*^∆K/∆K^ mice at 12 weeks of age in response to contractile agonists and vasodilators. The force developed in response to phenylephrine was significantly decreased in the thoracic aorta from *Myh11*^∆K/∆K^ mice compared to that in WT mice (p < 0.01, Fig. 4A). The maximum force developed in response to phenylephrine or potassium chloride treatment was significantly reduced in the *Myh11*^∆K/∆K^ aorta compared to the WT aorta (Fig. 4B). This reduced contractility in *Myh11*^∆K/∆K^ SMCs may contribute to the decreasing mechanoadaptation of the aortic wall. By contrast, the vasodilation functions in response to acetylcholine (for endothelium-dependent vasodilation) or nitroprusside (for endothelium-independent vasodilation) were comparable between the WT, *Myh11*^∆K/+^ and *Myh11*^∆K/∆K^ aortas (Fig. 4C).

### Ang II induced aortic dissections in *Myh11* K1256del mutant mice

**Figure 5:**
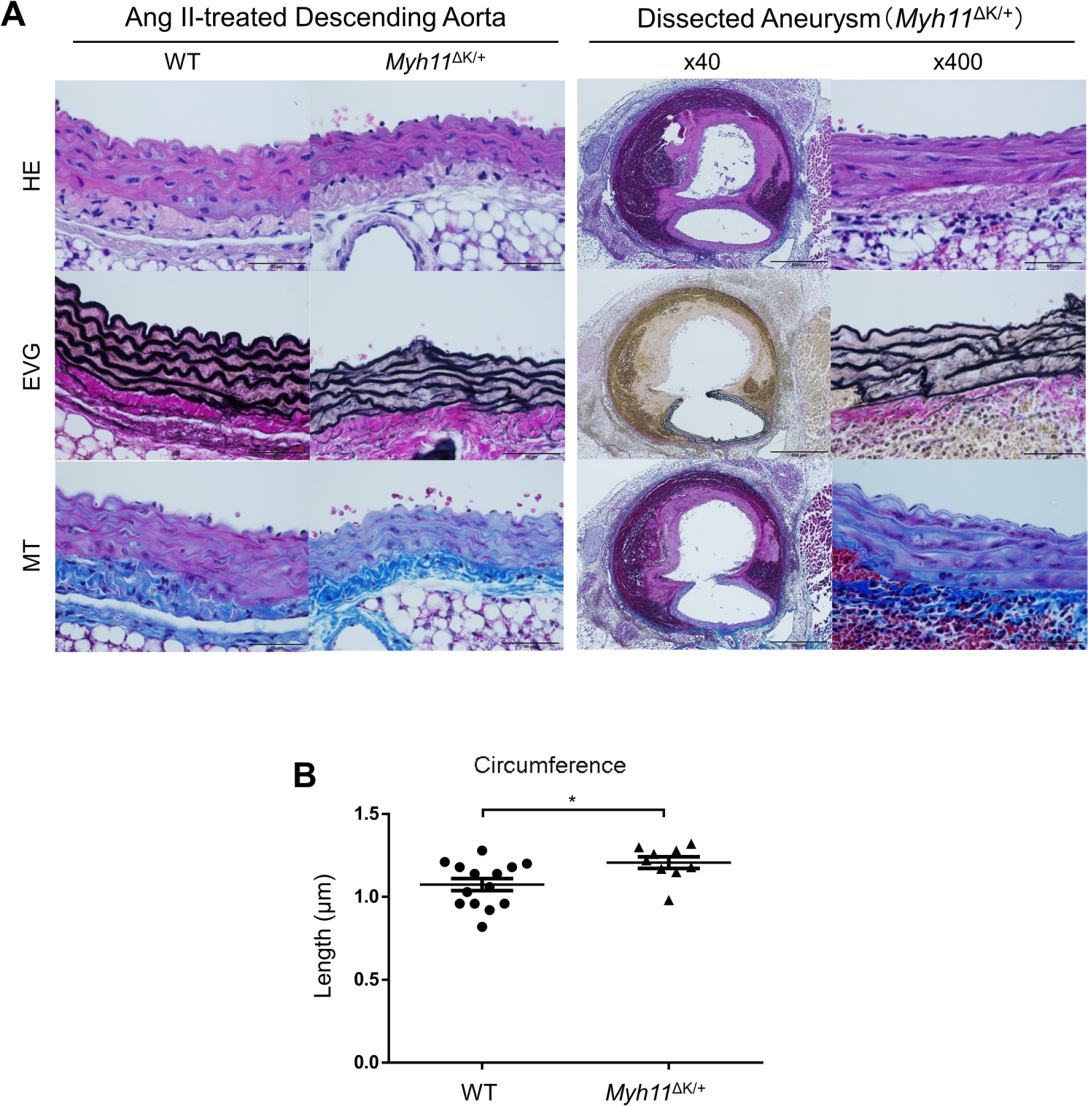
Angiotensin II (Ang II) infusion induces aortic dissection in *MYH11*^∆K/+^ mice.** (A)** Pathohistological images of descending aortas from Ang II-treated WT and *Myh11*^∆K/+^ mice. Cross-sections were stained with HE, EVG and MT (upper panel). The left part in the panel shows elastic fragmentation and fibrotic tissue deposition in non-dissected regions (scale bars = 50 µm, × 400). **(B)** Circumference of WT and *Myh11*^∆K/+^ ascending thoracic aortas after two weeks of Ang II infusion. WT: n = 14; *Myh11*^∆K/+^: n = 9. * p < 0.05, Mann–Whitney U test.

The attenuation of the structural intensity and maladaptation against mechanical stress in the mutant aortas may predispose people to developing thoracic aortic dissection. To investigate the pathological mechanisms of aortic dissection associated with *Myh11* K1256del, we administered Ang II (1000 ng/kg/min) to WT (n = 16) and *Myh11*^∆K/+^ (n = 15) males at eight weeks of age with osmotic pumps. Systolic blood pressure was measured before infusion pump implantation and after two weeks of Ang II treatment. Ang II-treated mice showed an increase in systolic blood pressure, but there was no significant difference between WT and *Myh11*^∆K/+^ mice (before implantations: p = 0.97, after treatments: p = 0.96; Supplementary Fig. 5A). Small intramural haematoma was frequently observed in the thoracic and abdominal *Myh11*^∆K/+^ aorta (Supplementary Fig. 5B). Aortic dissections occurred in six *Myh11*^∆K/+^ mice (40.0%), and two mice (13.3%) died of aneurysmal ruptures. There was no bias in the sites of aortic dissections between the thoracic and abdominal *Myh11*^∆K/+^ aortas (Supplementary Fig. 5C). In contrast, intramural haematoma or aortic dissections were not induced in Ang II-treated WT mice. We also attempted to generate a sufficient number of Ang II-treated *Myh11*^∆K/∆K^ mice for analysis, but it was difficult to obtain aortic samples because most mice died from aneurysmal ruptures. In a preliminary experiment, three of four Ang II-treated *Myh11*^∆K/∆K^ mice suddenly died in from two to four days after the start of the infusion. We dissected these mice and found a rupture in the ascending aorta or the aortic arch (data not shown). Histologically, Ang II-treated *Myh11*^∆K/+^ aortas showed a fragmentation of the elastic lamellae and fibrotic tissue deposition and displayed luminal expansion (Fig. 5A, B). Significant difference was not observed in the expression of the Ang II type 1 receptor (AGTR1) in the WT and *Myh11*^∆K/∆K^ aortas (Supplementary Fig. 5E).

### *Myh11* del1256K pathogenic variation does not alter the expression of smooth muscle myosin heavy chain isoforms and the expression or phosphorylation of proteins involved in smooth muscle contraction

**Figure 6:**
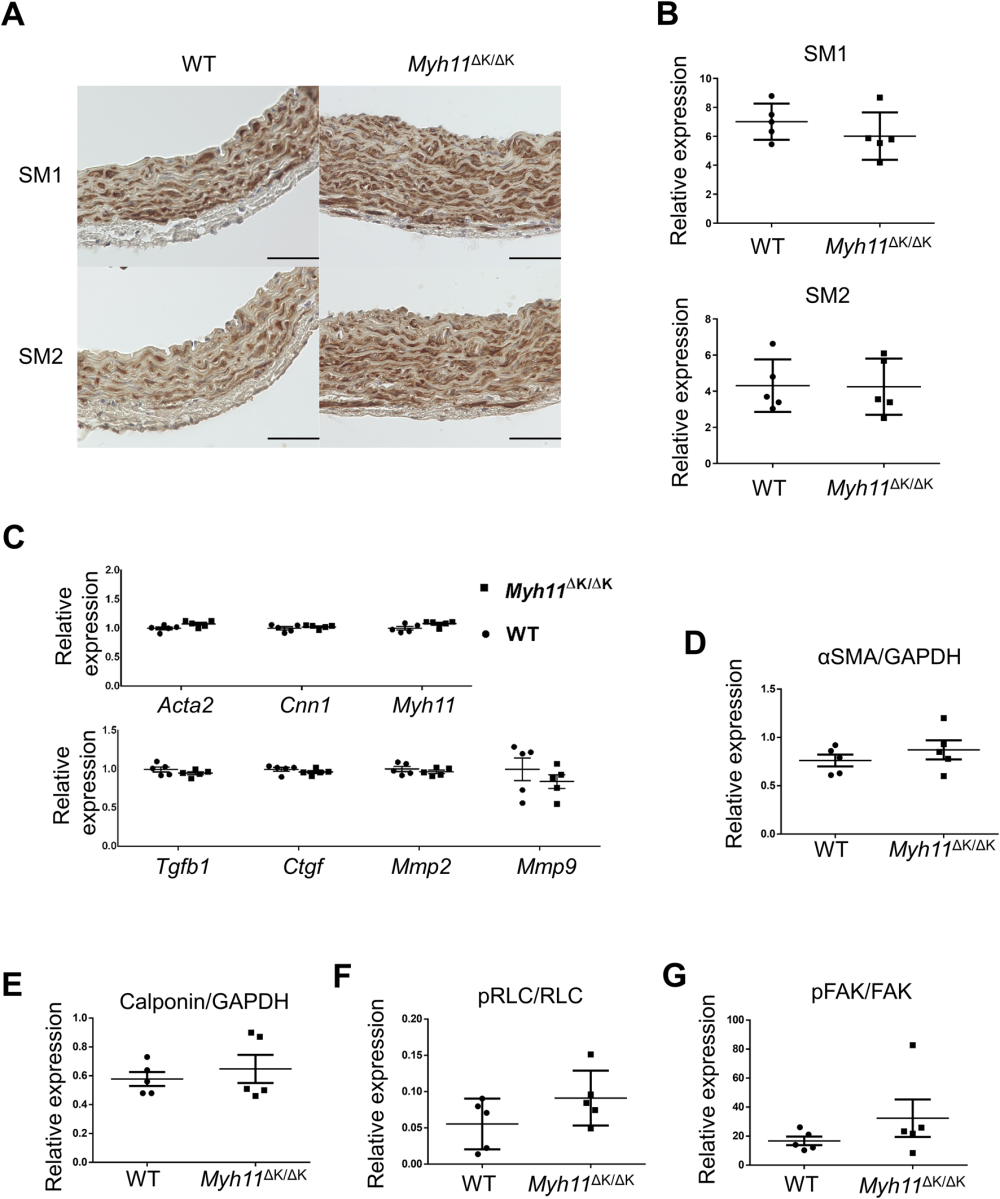
Expression and phosphorylation of proteins in *Myh11*^∆K/∆K+^ aortas.** (A)** Immunostaining for SM1 and SM2 showed no obvious differences in expressions and distributions between the WT and *Myh11*^∆K/∆K^ ascending aortas (scale bars = 50 µm, × 400). **(B)** Densitometric analysis of the expression of SM1 and SM2 in WT and *Myh11*^∆K/∆K^ aortas. Protein expression levels were normalised to that of GAPDH (n = 5). The Mann–Whitney U test showed that the difference was not statistically significant. **(C)** RT-qPCR analysis of *Acta2*, *Cnn1*, *Myh11*, *Tgfb1*, *Ctgf*, *Mmp2* and *Mmp9* mRNA isolated from thoracic aortas of WT and *Myh11*^∆K/∆K+^ mice at 12 weeks of age. Gene expression levels were normalised to *Gapdh*. Gene expression did not significantly differ between the WT and *Myh11*^∆K/∆K^ aortas (WT vs *Myh11*^∆K/∆K+^ n = 5, *Acta2*; p = 0.11, *Cnn1*; p = 0.73, *Myh11*; p = 0.06, *Tgfb1*; p = 0.41, *Ctgf*; p = 0.29, *Mmp2*; p = 0.53 and *Mmp9*; p = 0.41). **(D)** Densitometric analysis of the expression of αSMA in WT and *Myh11*^∆K/∆K^ aortas. Protein expression levels were normalised to that of GAPDH (n = 5). The Mann–Whitney U test showed that the difference was not statistically significant. **(E)** Densitometric analysis of the expression of calponin in WT and *Myh11*^∆K/∆K^ aortas. Protein expression levels were normalised to that of GAPDH (n = 5). The Mann–Whitney U test showed that the difference was not statistically significant. **(F)** Densitometric analysis of the ratio of phosphorylated myosin regulatory light chain (RLC) to total RLC expression in WT and *Myh11*^∆K/∆K^ aortas (n = 5). The Mann–Whitney U test showed that the difference was not statistically significant (p = 0.222). **(G)** Densitometric analysis of the ratio of phosphorylated focal adhesion kinase (FAK) expression to total FAK expression (n = 5). The Mann–Whitney U test showed that the difference was not statistically significant (p = 0.417).

We investigated the gene and protein expressions of aortas from WT and *Myh11*^∆K/∆K^ males at 12 weeks of age. Because the morphological features in mutant SMCs on electron micrograph suggests transformation from a contractile phenotype to a synthetic phenotype in SMCs, we analysed the expression of SM isoforms (SM1 and SM2) in the aortas, which indicate SMC differentiation^14–16^. However, we found that aortic smooth muscles does not undergo phenotypic modulation overall as seen in acute vascular injuries because the SM1 and SM2 expression in the mutant aorta did not show any abnormality compared to WT mice at 12 weeks of age (Fig. 6A, B and Supplementary Fig. 11). To further investigate whether the expression or phosphorylation of proteins involved in smooth muscle contraction is altered in *Myh11*^∆K/∆K^ SMCs, we measured the expression of these genes by RT-qPCR and protein levels by immunoblotting. However, no significant differences were noted between the WT and *Myh11*^∆K/∆K^ aortas in the expression of α-smooth muscle actin (*Acta2*), SM-MHC (*Myh11*) and calponin (*Cnn1*) or in the level of myosin regulatory light chain (RLC) phosphorylation (Fig. 6C–F and Supplementary Figs. 6A, 12). The phosphorylation level of focal adhesion kinase (FAK), a key molecule in the signalling cascades of focal adhesion, was also comparable between the WT and *Myh11*^∆K/∆K^ aortas (Fig. 6G and Supplementary Figs. 6A, 13, 14). The expression of a proliferation marker, proliferating cell nuclear antigen and Cyclin D1 were also comparable between the WT and *Myh11*^∆K/∆K^ aortas (Supplementary Fig. 6B). We also investigated the gene expression of TGF-ß (*Tgfb1*) and the transcriptional factors of its downstream cascade [connective tissue growth factor (*Ctgf*), MMP2 (*Mmp2*) and MMP9 (*Mmp9*)], which are associated with the pathogenesis in TAD, but no significant differences in their expression were observed between the WT and *Myh11*^∆K/∆K^ aortas (Fig. 6C).

### *Myh11* K1256del pathogenic variant impairs the stemness of induced pluripotent stem cells, but the stemness is maintained by reprogramming with Yamanaka factors plus Nanog

We endeavoured to identify the mechanism by which *Myh11* K1256 deletion results in aortic dissection *in vitro* by differentiating *Myh11*^ΔK/ΔK^-induced pluripotent stem cells (iPSCs) into SMCs. We first established iPSCs from mouse embryonic fibroblasts with the stable expression of Yamanaka factors (Oct3/4, Sox2, Klf4 and c-Myc) and evaluated the *Myh11*^ΔK/ΔK^ iPSCs on their comparability to WT iPSCs (Supplementary Fig. 7A)^17^. The number of alkaline phosphatase positive colonies did not differ significantly (Supplementary Fig. 7B, C). We then evaluated the pluripotency by generating embryoid bodies (EBs) by the hanging drop method and determined the tri-lineage differentiation of each genotype. As shown in the supplementary movie, the WT EBs produced beating cells. However, most of the cells that grew out of the *Myh11*^ΔK/ΔK^ EBs were non-beating and non-adherent, suggesting that the *Myh11* K1256del pathogenic variant impairs the maintenance of pluripotency. Each genotype had a similar morphology for nine days after retroviral transduction, but the *Myh11*^ΔK/ΔK^ colonies did not maintain their morphology, and granular cells started to appear at passage 3 (Supplementary Fig. 8A). We then transduced *Nanog* into the MEFs in addition to the Yamanaka factors. Surprisingly, the forced expression of *Nanog* along with the Yamanaka factors during somatic cell reprogramming allowed the *Myh11*^ΔK/ΔK^ iPSCs to maintain the ESC-like morphology (Supplementary Fig. 8B).

### Genes related to cell adhesion are downregulated in *Myh11*^ΔK/ΔK^ aortas and in SMC-lineage cells differentiated from iPSCs

**Figure 7:**
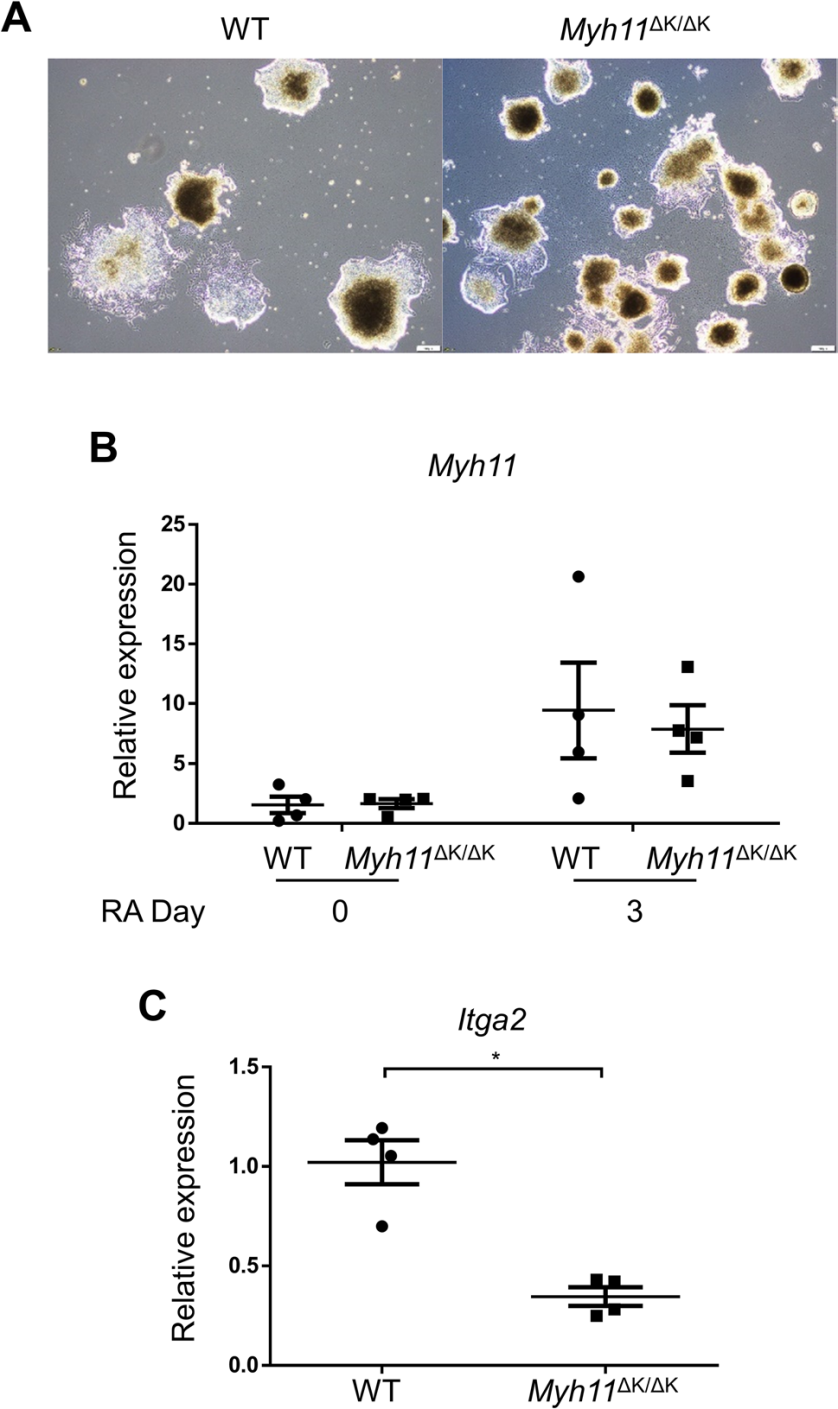
*Itga2* is downregulated in the SMC-lineage cells differentiating from *Myh11*^∆K/∆K^ iPSCs.** (A)** Phase-contrast images of WT and *Myh11*^∆K/∆K^ iPSCs at day 5 of differentiation by retinoic acid (scale bar = 200 µm). **(B)** RT-qPCR analysis of *Myh11* mRNA isolated iPSCs at days 0 and 3 of treatment with retinoic acid. Gene expression levels were normalised to18s rRNA (n = 4, day 0: p = 0.9714; day3: p > 0.9999). Mann–Whitney U test. RA = retinoic acid. **(C)** RT-qPCR analysis of *Itga2* mRNA isolated iPSCs at day 3 of differentiation by retinoic acid. Gene expression levels were normalised to18s rRNA. *p < 0.05, Mann–Whitney U test.

Nanog binds to an enhancer region of the human α-catenin gene (*Ctnna2*) (GeneCards website: http://www.genecards.org)^18^. The observation that iPSC stemness was improved by forced Nanog expression prompted us to examine the downregulated genes in the *Myh11*^ΔK/ΔK^ aortas we identified by RNA sequencing analysis for involvement in cell adhesion using the Gene Ontology (GO) database^19,20^. An examination of those genes for interactions with each other in the STRING database^21^ then revealed that *Ctnna2* had the highest number of interactions with the molecules related to intercellular adhesion (Supplementary Fig. 9). We further investigated the functional difference between WT and *Myh11*^ΔK/ΔK^ SMCs by inducing the differentiation of iPSCs into the SMC lineage by culturing the cells in media containing retinoic acid without the leukaemia inhibitory factor. At day 5 of differentiation, the cells had not assumed an SMC-like morphology (Fig. 7A). *Myh11* was upregulated regardless of the genotype at day 3 of differentiation (Fig. 7B), indicating the cells had committed to the SMC lineage. Integrin subunit α2 (*Itga2*) was downregulated in the aneurysmal aortas of SMC-specific *Smad4* knockout mice^22^, and a STRING protein–protein interaction analysis showed that *Itga2* had the second highest number of interactions with the focal-adhesion-related molecules that were downregulated in the *Myh11*^*ΔK/ΔK*^ aorta (Supplementary Fig. 9). Measurements of the mRNA expression of *Itga2* in the SMC-lineage cells also showed a significant reduction in *Itga2* expression in the *Myh11*^ΔK/ΔK^ cells (Fig. 7C).

## Discussion

We generated a novel mouse model of FTAAD with high reproducibility of aortic dissection and intramural haematoma by Ang II-treatment. We found that *Myh11* K1256del leads to structural fragility and maladaptation against mechanical stress in aortas by decreasing the composition of elastin lamellae and SMC contractility. This altered property of *Myh11* K1256del aortas increases association with the onset of aortic dissection.

We predict that myosin filament assembly may be interrupted in the presence of K1256del based on molecular structure modelling^23^. Myosin is typically divided into two fragments: N-terminal heavy-meromyosin (HMM) and C-terminal light-meromyosin (LMM)^24^. HMM consists of the globular motor domain (S1) and the coiled-coil region connecting S1 and LMM (S2). LMM forms a thick filament by intertwining with the LMMs of other myosin molecules. K1256 is located on the N-terminal side of LMM, and the amino-acid sequence around K1256 adopts a typical pattern of a heptad repeat composed of hydrophobic residues at the first (a) and fourth (d) positions (Supplementary Fig. 10A). These hydrophobic residues are required to form a coiled-coil structure by providing hydrophobic contacts between two heavy chains^25^. Supplementary Fig. 10B shows a structural model of the K1256-containing region (1226–1288 residues) of Myh11. The hydrophobic residues of WT, except for V1242, are at the interface between two heavy chains and contact each other, suggesting that this region can form a coiled-coil structure. In contrast, the hydrophobic residues located on the C-terminal side of K1256 are exposed to the outside by the K1256 deletion because the residues rotate 100° on the α-helix structure. This change in side-chain orientation likely disturbs the coiled-coil structure stably assembled by the hydrophobic contacts, which may partially affect HMM functions, such as thick filament formation.

Large-scale electron microscopic imaging is useful for comprehensively observing tissue sections; this is the first example of applying this method to analyse a luminal organ. We found a significant decrease in the area of elastic lamellae, suggesting a thinning of the elastic lamellae. As a previous study of FTAAD has shown, the thinning of elastic lamellae seems to be a common structural modification of aortas with the pathogenic variant that causes FTAAD^6^. Furthermore, by conventional transmission electron microscopy, we observed pathological features associated with cellular stress responses, such as debris and myelin figures. The expression and distribution of SM isoforms (SM1 and SM2), which are molecular markers of SMC differentiation^14–16^, showed no obvious changes in the *Myh11*^∆K/∆K^ mutant. This suggests that smooth muscle in *Myh11* K1256del does not undergo extensive phenotypic modulation.

In this study, aortic dissection and intramural haematoma developed in *Myh11*^∆K/+^ mice within two weeks of Ang II infusion. In our model, we observed a reduced contracting force of the aortic ring following stimulation with phenylephrine or KCl. This observation agrees with a previous report of attenuated SMC contractility induced by the deletion variant in the rod portion and in the motor domain of myosin^11^. Large-scale electron microscopy also indicated a reduction in the structural integrity of the aortic wall. Furthermore, the expression of the Ang II type 1 receptor in the *Myh11*^∆K/∆K^ aorta was not significantly different from that in the WT, suggesting the WT and *Myh11*^∆K/∆K^ mice had a comparable level of sensitivity to exogenous Ang II. A previous study has shown that applying calcium chloride to the aorta along with the Ang II infusion can cause aortic dissection in mice^26^. In the model, increased haemodynamic stress due to the stiffening of the aorta was proposed to cause dissection in aortas with reduced wall strength^26^. Thus, the amplified haemodynamic stress caused by attenuated contraction might have ultimately caused aortic dissection.

The stabilisation of the submembranous cytoskeleton is viewed as important for the efficient force development of SMCs, and two pathways of smooth muscle contraction have been proposed^27^. One pathway involves myosin RLC phosphorylation^27^, but our RLC phosphorylation analysis by immunoblotting did not indicate any attenuation of this pathway in *Myh11*^ΔK/ΔK^ aortas. The other pathway involves mechanotransduction at focal adhesion junctions^27^. Our RNA sequencing of the *Myh11*^ΔK/ΔK^ aorta showed a downregulation of the *Itga2* gene that encodes integrin subunit alpha 2 (Itga2), which is involved in focal adhesion^28^ (*Myh11*^ΔK/ΔK^ /WT ratio (log2) = − 1.76). Regardless of their genotypes, the iPSCs upregulated *Myh11* after culture with retinoic acid for three days, suggesting that K1256 deletion did not inhibit differentiation into SMCs. The expression of *Myh11* was comparable between the WT and *Myh11*^∆K/∆K^ cells, but the expression of *Itga2* was lower in the *Myh11*^∆K/∆K^ iPSCs than in the WT iPSCs. The newly differentiated cells showed a decrease in *Itga2* expression, indicating that the downregulation of *Itga2* detected in the *Myh11*^ΔK/ΔK^ aorta detected by RNA sequencing analysis is directly caused by the *Myh11* K1256del pathogenic variant rather than a secondary response to defects caused by the pathogenic variant. This implies a possible role of Myh11 in the modulation of focal adhesion through the regulation of Itga2 expression. Previous reports have indicated that polymorphism in *Itga2* is associated with ischemic stroke and coronary atherosclerosis^29^ and that *Itga2* is downregulated in the aortic aneurysm model induced by the inactivation of *Smad4*^22^. The present study is the first to report a downregulation of *Itga2* in the aorta in an FTAAD model.

Previous studies by our group as well as several other groups have shown that primary SMCs rapidly lose their original phenotypes when they are cultured in vitro^15,30–32^. Thus, in vitro studies of primary SMCs are not likely to represent in vivo events. To obtain cultured SMCs that better resemble tissue SMCs, we established iPSCs and induced the differentiation of the iPSCs into SMCs. The iPSCs that we generated from *Myh11*^ΔK/ΔK^ MEFs with the forced expression of Yamanaka factors also lost pluripotency and the ability to self-renew and to remain undifferentiated within five passages. Interestingly, the forced stable expression of Nanog, along with the Yamanaka factors, improved the stability of *Myh11*^ΔK/ΔK^ iPSCs. Since Nanog is known to bind to one of the enhancer regions of human α-catenin^18^, the abilities of iPSCs to self-renew, remain undifferentiated and maintain pluripotency might have been rescued by the upregulation of α-catenin and the subsequent stabilisation of intercellular adhesion. Our RNA sequencing data of the aorta also showed a decrease in the expression of *Ctnna2* (*Myh11*^ΔK/ΔK^ /WT ratio (log2) = − 10.8) as well as in a few other genes involved in intercellular adhesion. The STRING database showed that *Ctnna2* encoding α-catenin interacted with the highest number of the intercellular adhesion-related molecules that were downregulated in the *Myh11*^ΔK/ΔK^ aortas. Previous work has shown that α-catenin connects cadherin and the actomyosin network and that the loss of α-catenin function disrupts intercellular adhesion^33^. Moreover, α-catenin is known to act as a mechanosensor^33^ that enhances cell adhesion and promotes actin reorganisation at cell junctions^33^. In the future, a study using aortas either from *Ctnna2* knockout or overexpression mice may reveal the involvement of *α*-catenin in the stabilisation of the submembranous cytoskeleton and its contribution to aortic contraction. Furthermore, increasing the expression of α-catenin could be an effective intervention for improving intercellular adhesion and may be a novel strategy for the treatment of FTAAD.

Finally, mechanical signals are relayed by three mechanisms. Mechanical signals from (1) the focal adhesion are relayed to (2) the intercellular adhesion junction via (3) the actomyosin network^34^. From the intercellular adhesion junction, the signals are passed on to the neighbouring cells^34^. In this study, we found genetic and phenotypic change pointing towards defects in all three mechanisms in our FTAAD model. We showed the possibility of attenuated focal adhesion. *Itga2*, which is involved in focal adhesion, is downregulated in both *Myh11*^ΔK/ΔK^ aortas and in cells differentiating into the SMC lineage. Then, we obtained ultrastructural anomalies by large-scale electron microscopy suggestive of defective intercellular adhesion. In addition, the expression of genes, such as *Ctnna2* encoding α-catenin, that regulate intercellular adhesion was reduced. We predict, based on structural analysis (Supplementary Fig. 10), that the coiled-coil structure of the *Myh11* K1256del myosin II molecule is not optimal; therefore, this structure may attenuate the formation of the actomyosin network. Hence, we suspect that *Myh11* K1256del causes a defect in all three mechanisms mentioned above and that they all contribute to the reduced contractility of *Myh11*^ΔK/ΔK^ aortas. We point out the importance of testing for the integrity of all of the three mechanisms and monitoring the contractility of the SMC network rather than focusing on the dysfunction of individual smooth muscles. When we develop a new therapeutic strategy to improve the contractility of SMCs, it may be essential to target all of the three mechanisms.

Although the attenuation of SMC contractility did not lead to luminal expansion in the *Myh11*^∆K/∆K^ aorta, the uterus showed marked dilatation with a thinner wall. Furthermore, stillbirth was significantly more frequent with *Myh11*^∆K/∆K^ mothers compared to WT mothers, supporting that Myh11 plays a role in uterine contraction. We also observed PDA in all *Myh11*^∆K/∆K^ mice, whereas few *Myh11*^∆K/+^ mice exhibited PDA. The inadequate contraction of the ductus arteriosus in response to oxygen may induce PDA in *Myh11*^∆K/∆K^ mice. Compared with previously reported animal models with PDA which died within 24 h after birth^35–38^, most *Myh11*^∆K/∆K^ mice survived for more than 18 months with PDA, suggesting that these mice are a unique animal model of long survival with PDA.

In summary, *Myh11* K1256del induces pathogenic stress on the aortic wall through the induction of structural fragility and a disorder of mechanoadaptation in the aorta. These changes may be caused by defects in focal adhesion, intercellular adhesion and the actomyosin network. Further studies are necessary to understand the linkage between the abnormal LMM structure and the downregulation of genes that form focal adhesions and intercellular adhesions as this knowledge is critical for developing a preventive therapy for FTAAD.

## Methods

A detailed description of the methodology is provided in the Supplementary data.

### Animals

C57BL/6 J mice with the wild type or pathogenic variant in *Myh11* were kept under a 12-h light/dark schedule (lights on from 07:00 h to 19:00 h). Before invasive procedures such as the implantation of an infusion pump, the mice were anaesthetised by a single intraperitoneal injection of a mixture of medetomidine chloride (0.3 mg/kg), midazolam (4 mg/kg) and butorphanol tartrate (5 mg/kg)^39^. The absence of pedal reflex was used as the indicator of deep anaesthesia. Before sampling tissues from the mice, they were euthanised by a single intraperitoneal injection of an overdose of sodium pentobarbital (100 mg/kg). All animal handling procedures in this study complied with the Jichi Medical University Guide for Laboratory Animals, the Guide for the Care and Use of Laboratory Animals published by the U.S. National Institutes of Health (NIH Publication, eighth edition, 2011) and the ARRIVE guidelines^40^. The Institutional Animal Care and Concern Committee at Jichi Medical University approved all experimental protocols. With the exception of the histological analysis of the uterus, this study used male mice.

### Preparation of nickase Cas9 mRNA, gRNAs and *Myh11* Δ1256K DNA fragment

Nickase Cas9 [also known as Cas9 (D10A)^41^] mRNA was obtained using the mMESSAGE mMACHINE T3 kit (Ambion) and *Sap* I-linearised pBS-NFCas9 (D10A)A as an RNA synthesis template. This pBS-NFCas9 (D10A)A plasmid was constructed by replacing GAT [coding for aspartic acid (D) at the 10th amino acid of Cas9 protein] with GCT [coding for alanine (A)] in the pBS-NFCas9A plasmid^15^ using the inverse PCR method. The fidelity of the nucleotide sequence at the exchanged portion was confirmed by sequencing.

Guide RNAs (gRNAs) targeted to *Myh11* (NM_001161775.1) were prepared as described by Sakurai et al.^42^ and named gRNA1 and 2 (Fig. 1A and Supplementary Table 2). A 220-base pair *Myh11* donor DNA fragment, in which AAG (coding for lysine) at the 1256th amino acid of MYH11 protein was deleted, was constructed through a chemical gene synthesis method (TaKaRa Bio) and termed Δ1256K. In Δ1256K, the sequence corresponding to PAM was changed to sequences corresponding to those recognised by both gRNA1 and 2 (Fig. 1A and Supplementary Table 2). This Δ1256K fragment was then cloned into the pMD20 plasmid (TaKaRa Bio). Upon microinjection, the Δ1256K DNA donor fragment was removed from the plasmid backbone by digestion with *Bam*HI and *Nde*I.

### Generation of Myh11 1256 K-deficient mutant mice

To investigate the pathological effects of the deletion variant of *Myh11* 1256 K, we attempted to introduce this variant into B6 mice using the standard CRISPR-Cas9 system (gRNA 1 and Δ1256K DNA donor (Fig. 1A)), but this gene manipulation resulted in embryonic lethality. We did not further study the cause of embryonic lethality but speculated that it may be caused by the indels in *Myh11* leading to complete loss-of-function or off-target effects. Therefore, we used the CRISPR-nickase Cas9 system (two gRNAs and Δ1256K DNA donor (Fig. 1A)) to generate four founder mice (Fig. 1B): three heterozygotes (one male and two females) and one male homozygote (Fig. 1C, D). The heterozygous breeding pairs were used to generate homozygotes, but they failed to foster their pups for unknown reasons. The cause of neglect was not studied and remains unknown. The founder homozygote suddenly died at four weeks old (of uncertain cause but not aortic disease). We next collected sperm from the dead homozygote, which was used for in vitro fertilisation-embryo transfer with B6 females. As a result, we generated five heterozygotes of the next generation and backcrossed them more than three times. Subsequently, WT, heterozygous (*Myh11*^*∆K/*+^) and homozygous (*Myh11*^*∆K/∆K*^) mice obtained by line crossing were used for analysis. The genotypes of all mice were determined by a DNA sequence analysis of the genetically modified site (Fig. 1E and Supplementary Table 3).

### Histopathology

The excised aorta, bladder and uterus were fixed in 4% paraformaldehyde and embedded in paraffin. The paraffin-embedded tissues were sectioned into 5-μm-thick slices and stained with haematoxylin and eosin (HE), Elastica van Gieson (EVG) and Masson trichrome (MT). Morphometrical analysis was performed by using ImageJ/Fiji^43^. Immunostaining with anti-SM1 and anti-SM2 antibodies was performed as previously described^14^.

### Acquisition of large-scale electron microscopic images

Thoracic aortas were fixed with 2.5% glutaraldehyde in 0.1 mol/L phosphate buffer (PB, pH 7.4) followed by fixation in 1% osmium tetroxide in 0.1 mol/L PB. Samples were embedded in epoxy resin, cut into 70-nm-thick ultrathin sections and stained with uranyl acetate and lead citrate. Hundreds of digital images of compartmentalised rectangular areas covering entire transverse sections were acquired using a backscattered electron detector in scanning electron microscopy (JSM-7800F, JEOL). All images were stitched into a single tiling image, and large-scale tiling images were observed using a JavaScript-based viewer.

### Analysis of smooth muscle contraction

Approximately 10 mm of the aortic rings were removed from the anesthetised mice and attached with stainless-steel clips in an organ bath (20 mL) filled with a Krebs–Henseleit buffer, which was maintained at 37 °C and aerated with 95% O_2_/5% CO_2_ throughout the experiment. All ring strips were subjected to a 10-mN preload and incubated for 60 min. Contractions to subsequent phenylephrine administration were determined sequentially and corrected with tissue weight. The rate of relaxation was also obtained with subsequent acetylcholine and sodium nitroprusside administration after the peak of the contractile response to phenylephrine had been reached (10^–5^ mol/L).

### Aortic dissection model

Osmotic mini pumps (model Alzet 1002; DURECT Corporation) were implanted into the mice at eight weeks of age. Pumps were filled with Ang II solution (Peptide Institute, Inc.) by infusion at a rate of 1,000 ng/kg/min. Systolic blood pressure was measured before implantation and after two weeks of Ang II treatment. All mice surviving for two weeks were sacrificed, and their aortas were excised.

### Real-time quantitative polymerase chain reactions (RT-qPCR)

Total RNA extraction and RT-qPCR were conducted according to standard procedures. Primers for the various genes were designed, and the sequences are listed in Supplementary Table 4. Experiments were performed in triplicate, and all data were normalised to the expression of *Gapdh.*

### Immunoblot analysis

Protein lysates were prepared from the thoracic aorta. Next, 5 µg of total protein for each sample was separated on a 10% Bis–Tris gel or 3–8% Tris–Acetate gel (Thermo Fisher Scientific) by SDS-PAGE, electroblotted onto nitrocellulose membranes using the iBlot2 Dry Blotting System (Invitrogen) and immunoblotted with primary antibodies. Membranes were incubated with appropriate horseradish peroxidase-conjugated secondary antibodies overnight and visualised with a chemiluminescence kit (Bio-Rad Laboratories).

### Generation of molecular model of Myh11 protein

The models were constructed by amino-acid replacement with the crystal structure of human cardiac β-myosin S2 fragment (PDB id, 2FXO) as a template for the coiled-coil structure using the Molecular operating environment (MOE) program suite (Ver. 2016.08, Chemical Computing Group Inc., https://www.chemcomp.com).

### Statistical analysis

Data are expressed as the mean ± SEM. A Mann–Whitney U test was used to compare the distributed data between two different groups. Comparisons of multiple groups that passed a Kolmogorov–Smirnov test for normality were performed by one-way analysis of variance (ANOVA) with a Tukey post-hoc test. A p-value < 0.05 was considered significant, and a p-value < 0.01 was considered highly significant.

### Supplementary Information

Supplementary Information 1.

Supplementary Information 2.

Supplementary Video 1.

## Data Availability

The data underlying this article will be shared on reasonable request to the corresponding author.
