# Author Correction: An *Myh11* single lysine deletion causes aortic dissection by reducing aortic structural integrity and contractility

**DOI:** 10.1038/s41598-024-58098-4

**Published:** 2024-04-03

**Authors:** Keita Negishi, Kenichi Aizawa, Takayuki Shindo, Toru Suzuki, Takayuki Sakurai, Yuichiro Saito, Takuya Miyakawa, Masaru Tanokura, Yosky Kataoka, Mitsuyo Maeda, Shota Tomida, Hiroyuki Morita, Norifumi Takeda, Issei Komuro, Kazuomi Kario, Ryozo Nagai, Yasushi Imai

**Affiliations:** 1 Division of Clinical Pharmacology, Department of Pharmacology, Jichi Medical University, 3311-1 Yakushiji, Shimotsuke, Tochigi, 329-0498 Japan; 2 Division of Cardiovascular Medicine, Department of Medicine, Jichi Medical University, Tochigi, Japan; 3 Department of Cardiovascular Research, Shinshu University Graduate School of Medicine, Nagano, Japan; 4 Department of Cardiovascular Sciences, University of Leicester Cardiovascular Research Centre, Glenfield Hospital, Leicester, UK; 5 System Integration Center, Gunma University Hospital, Gunma, Japan; 6 Department of Applied Biological Chemistry, Graduate School of Agricultural and Life Sciences, The University of Tokyo, Tokyo, Japan; 7 RIKEN Center for Biosystems Dynamics Research, Kobe, Japan; 8 RIKEN-JEOL Collaboration Center, Kobe, Japan; 9 Department of Cardiovascular Medicine, Graduate School of Medicine, The University of Tokyo, Tokyo, Japan; 10 Jichi Medical University, 3311-1 Yakushiji, Shimotsuke, Tochigi, 329-0498 Japan

Correction to: *Scientific Reports* 10.1038/s41598-022-12418-8, published online 25 May 2022

The Supplementary Information 2 file published with this Article contained an error, where the Supplementary Figures were not made available.

This error has now been corrected in the Supplementary Information 2 file that accompanies the original Article.

