# Supplementary material for: An Myh11 single lysine deletion causes aortic dissection by reducing aortic structural integrity and contractility
Source: Sci Rep. 2022 May 25;12:8844. doi: 10.1038/s41598-022-12418-8 (PMC9133116; doi:10.1038/s41598-022-12418-8)
Supplement: Supplementary file 2 — Supplementary Information 2. [file 41598_2022_12418_MOESM2_ESM.pdf]

Supplementary data

An Myh11 single lysine deletion causes aortic dissection by reducing aortic structural integrity and contractility

Keita Negishi[1,2†]; Kenichi Aizawa[1†], Takayuki Shindo[3], Toru Suzuki[4], Takayuki Sakurai[3], Yuichiro Saito[5], Takuya Miyakawa[6], Masaru Tanokura[6], Yosky Kataoka[7,8], Mitsuyo Maeda[7,8], Shota Tomida[1], Hiroyuki Morita[9], Norifumi Takeda[9], Issei Komuro[9], Kazuomi Kario[2], Ryozo Nagai[10]*, and Yasushi Imai[1]*

[1] Division of Clinical Pharmacology, Department of Pharmacology and [2] Division of Cardiovascular Medicine, Department of Medicine, Jichi Medical University, Tochigi, Japan

[3] Department of Cardiovascular Research, Shinshu University Graduate School of Medicine, Nagano, Japan

[4] Department of Cardiovascular Sciences University of Leicester Cardiovascular Research Centre, Glenfield Hospital, Leicester, UK

[5] System Integration Center, Gunma University Hospital, Gunma, Japan

[6] Department of Applied Biological Chemistry, Graduate School of Agricultural and Life Sciences, The University of Tokyo, Tokyo, Japan

[7] RIKEN Center for Biosystems Dynamics Research, Kobe, Japan

[8] RIKEN-JEOL Collaboration Center, Kobe, Japan

[9] Department of Cardiovascular Medicine, Graduate School of Medicine, The University of Tokyo, Tokyo, Japan

[10] Jichi Medical University, Tochigi, Japan

†These authors contributed equally to this work

*To whom correspondence should be addressed

Supplementary Methods

Histological and immunohistochemical studies. After perfusion with phosphate-buffered saline (PBS, pH 7.4), the aorta and bladder were excised from anesthetised mice. The uterus was also excised from females at the oestrous cycle stage identified by using stained vaginal smears. These tissues were fixed in 4% paraformaldehyde/0.1 mol/L phosphate buffer (PB, pH 7.4) at 4 °C for 24 h. The fixed tissues were dehydrated through an ethanol series, cleared with xylene, embedded in paraffin and sectioned into 5-μm-thick slices with a rotary microtome (MICROM HM360, ZEISS). The tissue sections were deparaffinised with xylene and rehydrated through an ethanol series and stained with haematoxylin and eosin (HE) to evaluate general morphology. Elastica van Gieson (EVG) and Masson trichrome (MT) staining were performed to specifically highlight elastic lamellas and collagen fibres. All staining reagents were purchased from Muto Pure Chemicals Co., Ltd., and staining was performed according to the manufacturer's protocol. The sections were examined with a BX63 Upright Microscope system (Olympus) at ×40, ×200 and ×400 magnifications. The circumference, the cross-sectional areas of the media and adventitia and the thickness of the myometrium were measured with ImageJ/Fiji software,[1] and their average thicknesses were calculated by dividing the area by the aortic circumference.

For the immunohistochemistry analysis of smooth muscle myosin heavy chain isoforms (SM1 and SM2), the aortas were harvested and fixed in 95% ethanol with 1% acetate acid for 24 h at 4 °C. Paraffin-embedded sections were prepared as described above. Endogenous peroxidase activity was blocked with 3% H_2O_2/methanol for 10 min at RT,

followed by incubation with 2.5% normal goat serum (S-1012, Vector Laboratories) for 20 min at RT. The sections were incubated with a primary antibody against SM1 (dilution 1:500; BM20, Kyowa Medix) for 60 min at RT, followed by incubation with peroxidase micropolymers (MP-7404, Vector Laboratories). SM2 antibody (7601, Yamasa Corporation) was labelled with horseradish peroxidase by using an HRP Conjugation Kit (ab102890, Abcam) in advance. Sections were incubated with the primary antibody labelled with HRP for 2 h at RT. DAB (SK-4105, Vector Laboratories) was used for detection, and slides were counterstained with haematoxylin.

Acquisition of large-scale electron microscopic images. Mice were perfused transcardially with fixative solution containing 2.5% glutaraldehyde and 4% sucrose in 0.1 mol/L PB. Thoracic aortas were removed, immersed in fixative solution for 2 h at 4 °C and then post-fixed with 1% osmium tetroxide in 0.1 mol/L PB for 90 min at 4 °C. Fixed tissues were dehydrated in graded ethanol and embedded in epoxy resin. The entire transverse ultrathin sections of the thoracic aorta of 70-nm thickness were cut, mounted on pieces of silicon wafers and contrasted with uranyl acetate and lead citrate. Large-scale electron microscopic images including entire transverse sections of the thoracic aortas were acquired using a backscattered electron detector with a scanning electron microscope (JSM-7800F, JEOL). Digital images (magnification ×2,000) of hundreds of compartmentalised rectangular areas covering each entire tissue section were obtained in a sequential order by moving a motorised stage of the specimens with merging areas of 15% of the next image, followed by the stitching procedure of all images into a single tiling image. The Grid/Collection

stitching plug-in in ImageJ/Fiji[1] was used to construct large-scale tiling images.[2] Large-scale electron microscopic images were observed using the JavaScript-based RIKEN CLST Electron Microscopic Viewer.[3]

Morphometric analysis of large-scale electron microscopy. Longitudinally sectioned SMCs (> 30 µm of cellular diameter and > 5 µm of nuclear diameter) were randomly selected from whole aortic ring sections obtained by large-scale electron microscopic images. The length (µm) of the adhesive surfaces between the selected cell and other adjacent cells was measured by using a dedicated ImageJ/Fiji.[1] The sum of each adhesive length was considered to indicate the strength of cell adhesion of a single SMC (Supplemental Fig. S4A).

The whole aortic ring image was parted into 50–60 sections at lengths of 50 µm. These 50-µm aortic sections were randomly selected, and elastic lamellae identified as regions of plain texture were marked in black by Photoshop CC (Adobe, Supplemental Fig. S4B) because ImageJ/Fiji[1] could not distinguish elastic fibres and other components of the ECM, such as collagens and proteoglycans, by the differences in the image texture. The number of pixels was measured in the elastic region, and aortic sections were selected by using ImageJ/Fiji[1] and were used to calculate the area ratio of the elastic lamellae.

Analysis of smooth muscle function. Approximately 10 mm of aortic rings were removed from anesthetised mice at 12 weeks of age and attached with the stainless-steel clips of the apparatus for isometric force measurement (RMT-1000; Nihon Kohden). The aortic rings

were mounted in an organ bath (20 mL) filled with Krebs–Henseleit buffer (K3753, Sigma-Aldrich), which was maintained at 37 °C and aerated with 95% O_2/5% CO_2 throughout the experiment. All ring strips were subjected to a 10-mN preload and incubated for 60 min. The aortic rings were contracted in the presence of 10–5 mol/L phenylephrine (Phe) for three contraction and relaxation cycles. The contractions to the subsequent Phe (10^{-10}–10^{-5} mol/L; 163-11791, Wako Pure Chemical Corporation) were determined sequentially and corrected with tissue weight (mg). The rate of relaxation was also obtained with subsequent acetylcholine (10^{-10}-10^{-5} mol/L; A6625, Sigma-Aldrich) and sodium nitroprusside (SNP 10^{-10}–10^{-5} mol/L; 1614501, Sigma-Aldrich) after the peak of the contractile response to Phe (10^{-6} mol/L). The measured isometric force was analysed with LabChart version 8.1.9 (BIO Research Center Co., Ltd.).

Aortic dissection model. At the age of eight weeks, wild-type (WT) and heterozygous (*MYH11*$^{\Delta K/+}$) mice were implanted with osmotic pumps (model Alzet 1002; DURECT Corporation) filled with a solution of angiotensin II (Ang II; Peptide Institute, Inc.) in saline, according to a previously published method with some modification.[4] The concentration of Ang II was adjusted so that it was infused at a rate of 1,000 ng/kg/min. Before the implantation of osmotic pumps, the mice were administered anaesthetic. The absence of pedal reflex was used as an indicator of deep anaesthesia. Pumps were placed into the subcutaneous space at the back of the neck. After implantation, the mice were allowed to rest on a heating pad until recovering and becoming alert. The mice were observed for two weeks to monitor their survival and incidence rates. Mice surviving for

two weeks were sacrificed by intraperitoneal injection of 100 mg/kg pentobarbital, and their aortas were excised. The aortas were photographed at 6.3× magnification with a camera (MC170 HD, Leica Microsystems) mounted on a stereo-microscope (M60, Leica Microsystems). The mice were adapted to the blood pressure system (MK-2000ST, Muromachi Kikai Co., Ltd.) at least three times before implantation and after two weeks of treatment with Ang II.

RNA extraction and real-time quantitative PCR (RT-qPCR). Total RNA from the thoracic aorta was extracted with RNeasy Tissue Fibrous Kit (74704, Qiagen) according to the manufacturer’s protocol. cDNA was synthesised from total RNA using ReverTra Ace with gDNA Remover (FSQ-301, TOYOBO), and endogenous genomic DNA was degraded by DNase I (Qiagen). qPCR was performed with a two-step cycling protocol (95 °C for 30 s followed by 40 cycles of 95 °C for 5 s and 60 °C for 30 s) or a three-step cycling protocol (95 °C for 1 min followed by 45 cycles of 95 °C for 15 s, 56 °C for 20 s and 72 °C for 45 s) by using SYBR Green PCR Master mix (Applied Biosystems) and LightCycler 480 (Roche Diagnostics). The primers for RT-PCR are listed in Supplemental Table S3. Experiments were performed in triplicate, and all data were normalised against the expression of *Gapdh*.

Immunoblot analysis. The excised aorta and bladder were trimmed of surrounding tissues and homogenised by using Bioprep-24 (Hangzhou Allsheng Instruments Co., Ltd.) in protein extraction solution (T-PER buffer [#78510, Thermo Fisher Scientific], protease inhibitor cocktail [1 tablet/10 mL; Roche Diagnostics] and phosphatase inhibitor cocktail [1

tablet/10 mL; Roche Diagnostics]). After homogenisation, the tissue lysates were centrifuged at 10,000 rpm for 10 min at 4 °C. The supernatants were collected and stored at -20 °C. Total protein concentrations were measured with the bicinchoninic acid Protein Assay Kit (Pierce Biotechnology). Protein (5 μg) for each sample was separated on a 10% Bis-Tris gel or 3–8% Tris-Acetate gel (Thermo Fisher Scientific) by SDS-PAGE and electroblotted to nitrocellulose membranes using iBlot2 Dry Blotting System (Thermo Fisher Scientific). The membranes were blocked with 5% skim milk in Tris-buffered saline with Tween (TBS-T; Cell Signaling Technology) for 1 h, washed and incubated overnight at 4 °C in TBS-T with primary antibodies. Target molecules were detected with the appropriate HRP-conjugated secondary antibody (Cell Signaling Technology) and chemiluminescence kit (#170-5060, Bio-Rad Laboratories). Signals were detected by using LAS-3000 (FUJIFILM), and quantitative analysis was performed using ImageGauge Version 4.23 (FUJIFILM). Primary antibodies include anti-α-smooth muscle actin, anti-calponin, anti-E-cadherin, anti-FAK (Cell Signaling Technology), anti-SM-MHC (Santa Cruz Biotechnology), anti-elastin (Bioss Antibodies) and anti-GAPDH (Ambion). Antibodies against E-cadherin and FAK were diluted with the signal enhancer (DRC-W00H250, DRC Co., Ltd.).

RNA-seq analysis. Thoracic aortas were dissected from WT, *Myh11*$^{\Delta K/+}$ and *Myh11*$^{\Delta K/\Delta K}$ mice. Total RNA was extracted using the RNeasy Tissue Fibrous Kit (74704, Qiagen) and the RNeasy MinElute Cleanup Kit (28004, Qiagen) according to the manufacturer's instructions. The RNA concentration and purity were measured with a Nanodrop instrument

(ND-1000, Thermo Fisher Scientific). The length of the RNA fragments was determined with an Agilent 2100 Bioanalyzer (Agilent, USA). The cDNA library was then prepared from 0.3μg of total RNA with oligo dT primer by reverse transcription followed by PCR amplification (TruSeq Standard mRNA Sample Prep Kit, Illumina) according to the manufacturer's protocol. The quality of the cDNA from aortas was assessed with a bioanalyzer (DNA 1000 Assay, Agilent), and the concentrations of the cDNA samples were determined by qPCR. Equal amounts of nine samples were pooled to prepare a 9-plex RNA-seq library. Single-end 50-base-pair reads were obtained from the 9-plex RNA-seq library. Using one lane of a flow cell of a HiSeq2500 instrument, the resulting sequence reads were then separated according to the index sequence. After the separation, the reads were trimmed and filtered using the MOIRAI workflow and mapped on the mm10 mouse genome assembly using STAR.[5]

Protein-protein interaction analysis. The genes downregulated in the *Myh11*$^{\Delta K/\Delta K}$ aorta according to RNA sequencing analysis were first filtered for involvement in the integrin or cadherin pathway by GO enrichment analysis[6] and AmiGo.[7] The genes identified as associated with the integrin or cadherin pathway were then inputted into the STRING database for incorporation into a protein-protein interaction diagram.[8,9,10,11,12,13,14,15,16]

Cell culture and retroviral infection. Retroviral vectors were prepared by transfecting pMXs-Oct4, Sox2, Klf4, c-Myc or Nanog into platinum-E packaging cells, as previously described.[17] Primary mouse embryonic fibroblasts were infected with the retroviral vectors.

The pMXs vectors were obtained from Addgene (https://www.addgene.org/). The iPSCs were routinely co-cultured with MEF or SNL feeder cells treated with mitomycin C (Fujifilm) on gelatine-coated dishes in Dulbecco's modified Eagle's medium (DMEM)/F12 (Thermo Fisher Scientific) in the presence of 20% foetal bovine serum (FBS; Hyclone or Biocera), 0.1mmol/L 2-mercaptoethanol, GlutaMAX (Thermo Fisher Scientific), nonessential amino acids, penicillin/streptomycin and 1000U mouse LIF (Fujifilm). Trypsin or collagenase was used to detach iPSCs before being reseeded for passage.

Embryoid body formation. The iPSCs were suspended in the embryonic fibroblast medium (EFM) consisting of Dulbecco's modified Eagle medium with 4300 mg/L glucose (Fujifilm), 10% foetal bovine serum (Hyclone), 1 mmol/L or 0.1 mmol/L 2-mercaptoethanol, GlutaMAX (TFS) and penicillin/streptomycin (Thermo Fisher Scientific). The cell suspension was pipetted on the inner side of the lid of a culture dish. Then, the lid was placed back on the culture dish to allow the cells to be incubated for 48 hours.

Statistical analysis. Data are expressed as the mean ± SEM unless otherwise indicated. 'n' represents the number of mice. A p-value < 0.05 was considered significant (*), and a p-value < 0.01 was considered highly significant (**). Statistical analysis and a graph drawing were performed with GraphPad Prism version 6.0.3 (GraphPad, Inc.). Comparisons of body weight between multiple groups were performed using one-way ANOVA with a Tukey post-hoc test. For RT-qPCR, immunoblotting, functional analysis

and morphometric analysis, comparisons between two different groups were calculated using a Mann-Whitney U test because of the small sample size. A chi-squared test was performed to compare the incidence of dissecting aneurysms in the Ang II infusion models.

Supplementary Tables

Supplementary Table 1. Occurrence of dissection in each aortic region. The ascending, descending and abdominal aorta of each mouse was examined for the presence of dissection (Fisher's exact test, p = 1.00).

Aortic region	Aortas examined	Aortas with dissection
Ascending	7	3
Descending	7	4
Abdominal	7	3

Supplementary Table 2. Sequences corresponding to PAM recognised by both gRNA1 and 2

ID	Sequences (5'-> 3')	Location	GeneBank #
gRNA1	GTTCCACCTCCTGCTTCGCCTGG	Myh11 exon 28	NM_001161775.1
gRNA2	GAAGCTGGAGGTGCAGCTGCAGG	Myh11 exon 28	NM_001161775.1
Myh11 (Δ1256K)	GGATCCGATTAGCAAGCAGACACTGG<br>AGAAGGAGAACGCGGACCTGGCTGG<br>GGAGCTGCGTGTCCTGGGCCAGGCG<br>AAGCAGGAGGTGGAACACAAGAAG<br>AAGCTGGAGGTGCAGCTGCAAGATC<br>TGCAGTCCAAGTGCAGTGATGGGGA<br>GCGTGCCCGGGCTGAGCTCAGTGAC<br>AAGGTCCACAAGCTACAGGTGAGGG<br>CAGGCAGCACAATCCATATG	Myh11 exon 28	NM_001161775.1

Supplementary Table 3. PCR primers for genotyping *Myh11* K1256del mice

ID	Sequence (5’ -> 3’)	Product size
Myh11ch-1S	GTTCTATGCACCCCTCCTCCCTTCC	525bp
Myh11ch-1A	TGTGACCAGCTCTCCCACTGATTACA	

Supplementary Table 4. qPCR primer sequences

Target	Forward sequence (5’-> 3’)	Reverse sequence (5’-> 3’)
Acta2	AGTGTGATATTGACATCAGGAAGGA	ACAGAGTACTTGCGTTCTGGAG
Cnn1	TGTCTGTGTCATCTGCACCTC	CACTCTCTCAGCTCCTGCTC
SM1+2	AGCCAAGCTCAAGTCCACTG	ATCTTGCGCTCATCCTCCAC
SM2	TGGAAGAGGCAGAGGAGGAG	TCTCCTGGTGGCTCACTG
Eln	ATTCCCAGGTGGAGGAGTTG	ACTTTCTCTTCCGGCCACAG
Gapdh	TGTGTCCGTCGTGGATCTGA	TTGCTGTTGAAGTCGCAGGAG
Sox2	GCACATGAACGGCTGGAGCAACG	TGCTGCGAGTAGGACATGCTGTAGG
Itga2	GGCTATGACTGCAGGTTTGTGGAAGCTCGG	GTTGAGTTCTGTGGTCTCATCCATCTCATC
18s rRNA	GTAACCCGTTGAACCCCATT	CCATCCAATCGGTAGTAGCG

Supplementary Figure Legends

Supplementary Figure 1. Low fertility in *Myh11*$^{\Delta K/\Delta K}$ female mice. (A) The number of pups at wean derived from the breeding pairs with *Myh*11$^{\Delta K/+}$ males (WT mothers, n = 27; *Myh11*$^{\Delta K/+}$ mothers, n = 27, *Myh11*$^{\Delta K/\Delta K}$ mothers, n = 17). Pups from *Myh11*$^{\Delta K/\Delta K}$ mothers frequently died during the neonatal period (pups from WT mothers, 6.8 ± 0.28; pups from *Myh11*$^{\Delta K/+}$ mothers, 5.4 ± 0.34; pups from *Myh11*$^{\Delta K/\Delta K}$ mothers, 1.5 ± 0.51). ** $P < 0.01$, one-way ANOVA. **(B)** Dead *Myh11*$^{\Delta K/\Delta K}$ mothers during delivery. **(C)** Body weights of 4-, 8-, 12- and 32-week-old male and female mice did not differ among genotypes (n = 20).

Supplementary Figure 2. *Myh11* K1256del descending aorta shows thickened media and adventitia. (A) Whole images of cross-sectional aortas (ascending, descending and abdominal) from 12-week-old WT, *Myh11*$^{\Delta K/+}$ and *Myh11*$^{\Delta K/\Delta K}$ mice. Cross-sections were stained with haematoxylin and eosin (HE), Elastica van Gieson (EVG, for elastin), Masson trichome [MT, for collagen; scale bars = 500 µm (×40), 200 µm (×100)]. Aortas with the pathogenic variant showed increased thickness of media and adventitia. **(B)** Morphometric parameters of WT, *Myh11*$^{\Delta K/+}$ and *Myh11*$^{\Delta K/\Delta K}$ descending thoracic aortas. ** $p < 0.01$, One-way ANOVA with Tukey post-hoc test.

Supplementary Figure 3. Enlarged bladder and hypoplastic uterus observed in *Myh11*$^{\Delta K/\Delta K}$ mice. (A) Enlarged bladder, swollen kidneys and hypoplastic uterus were observed in *Myh11*$^{\Delta K/\Delta K}$ mice at 12 weeks of age (scale bars = 1 cm). **(B)** *Myh11*$^{\Delta K/\Delta K}$ bladders showed non-uniform staining such as punch-out and gaps between smooth muscle

layers. **(C)** Smooth muscle layers in *Myh11*$^{ΔK/ΔK}$ uteruses were thinner than those in WT. Cross-sections were stained with haematoxylin and eosin (HE) and Masson trichome [MT, scale bars = 500 µm (×40), 200 µm (×100), 50 µm (×400)]. **(D)** Comparison of the thickness of the smooth muscle layer (myometrium) from the WT and *Myh11*$^{ΔK/ΔK}$ uteri (n = 4). The data are expressed as the mean ± SEM. * p < 0.05, Welch's T test.

Supplementary Figure 4. Procedures of morphometric measurements in the large-scale electron microscopic images. (A) 15 longitudinally sectioned SMCs (> 30 µm of cellular diameter and > 5 µm of nuclear diameter) were randomly selected from each aortic sample (WT and *Myh11*$^{ΔK/ΔK}$, n = 3). Next, the length of adhesive surfaces between the target cell and other adjacent cells was measured, and the sum of each adhesive length was considered to indicate the strength of cell adhesion of single SMC. **(B)** 15 50-µm sections from a whole aortic ring section (WT and *Myh11*$^{ΔK/ΔK}$, n = 3) were randomly selected. The area of plain texture in the sections is marked as elastic lamellae, and the ratio to the elastic area was calculated. The ratio of elastic area indicated the amount of elastic fibres in the aortic sections.

Supplementary Figure 5. Ang II enhanced medial degeneration in *Myh11*$^{ΔK/+}$ aorta, resulting in aortic dissections. (A) Ang II-treated groups of WT and *Myh11*$^{ΔK/+}$ mice showed increased systolic blood pressure as Ang II induced hypertension, but there was no significant difference between WT and *Myh11*$^{ΔK/+}$ mice [before Ang II-treatment (day 0): WT, 106.2 ± 3.0 versus *Myh11*$^{ΔK/+}$, 101.5 ± 2.75 mmHg, p = 0.97; after Ang II-treatment

(day 14): WT, 153.5 ± 3.2 versus *Myh11*$^{\Delta K/+}$, 150.6 ± 3.4 mmHg, p = 0.96]. **(B)** Small intramural hematoma was frequently induced in Ang II-treated *Myh11*$^{\Delta K/+}$ aorta. **(C)** Aortic dissections were induced not only in abdominal aortas but also in thoracic aortas of Ang II-treated *Myh11*$^{\Delta K/+}$ aorta. **(D)** Survival curves of the WT, *Myh11*$^{\Delta K/+}$ and *Myh11*$^{\Delta K/\Delta K}$ mice infused with angiotensin II (Ang II) for 14 days. WT vs *Myh11*$^{\Delta K/+}$: p = 0.0528; WT vs *Myh11*$^{\Delta K/\Delta K}$: p < 0.0001, Log-rank test. **(E)** Scatter dot plot showing the means ± SD of Ang II type 1 receptor expression normalised with GAPDH expression at baseline (n = 5). The Mann-Whitney U test showed that the difference was not statistically significant.

Supplementary Figure 6. Immunoblot analysis of proteins associated with contraction, cell adhesion and proliferation. (A) Immunoblot analysis of proteins associated with contraction and cell adhesion in thoracic aortas of WT and *Myh11*$^{\Delta K/\Delta K+}$ mice at 12 weeks of age. **(B)** Densitometric analysis of the expression of proliferating cell nuclear antigen (PCNA) (p = 0.8016) and cyclin D1 (p = 0.9444) in WT and *Myh11*$^{\Delta K/\Delta K}$ aortas. Protein expression levels were normalised to that of GAPDH (n = 5). The Mann-Whitney U test showed that the difference was not statistically significant.

Supplementary Figure 7. *Myh11*$^{\Delta K/\Delta K}$ iPSCs quickly lose the abilities to self-renew and remain undifferentiated. (A) Primary WT and *Myh11*$^{\Delta K/\Delta K}$ mouse embryonic fibroblast culture 24 h before transduction. **(B)** Alkaline phosphatase (AP) staining of colonies of from WT *Myh11*$^{\Delta K/\Delta K}$ cells after transduction of Oct4, Sox2, Klf4 and c-Myc (AP, Stem TAG, Cell Bio Inc.). **(C)** Scatter dot plots showing the average number of AP-positive

colonies per culture dish ± SEM. ImageJ/Fiji was used to count AP-positive colonies. **(D)** Phase-contrast images of WT and *Myh11*$^{\Delta K/\Delta K}$ cells outgrowing from embryoid bodies (EBs). The images were taken 7 days after EBs were plated on the gelatine-coated dishes. (scale bar = 200μm). *Myh11*$^{\Delta K/\Delta K}$ EBs did not produce any beating cells. Refer to the supplementary movie to see beating WT cells.

Supplementary Figure 8. Stemness of *Myh11*$^{\Delta K/\Delta K}$ is recovered by Nanog transduction along with the Yamanaka factors. (A) Phase-contrast images of iPSC colonies. The images were taken at passage (P) 0, P3 and P5 (scale = 80 μm, 800 μm and 800μm, respectively). **(B)** Phase-contrast images of WT and *Myh11*$^{\Delta K/\Delta K}$ iPSCs on gelatine-coated dishes at the 16th to 19th passage (scale bar = 200 μm).

Supplementary Figure 9. Protein-protein interaction network analysis of genes associated with the integrin or cadherin pathways. Proteins known to interact or be co-expressed with each other are connected with a line. A thicker line indicates a higher confidence of association. The red and blue node colours indicate involvement of the proteins in adherens junctions and focal adhesions, respectively. The diagram was drawn with the STRING database.

Supplementary Figure 10. Coiled-coil formation of the K1256-containing region of *MYH11*. (A) Amino-acid sequence of the 1226–1288 residues of *Myh*11. K1256 and hydrophobic residues were coloured in magenta and orange, respectively. The letters under

the sequence (abcdefg) indicate the order of heptad repeat. **(B)** Structural models of the 1226–1288 residues of *Myh11* (WT) and its ΔK1256 pathogenic variants (*MYH11*$^{\Delta K/+}$ and *MYH11*$^{\Delta K/\Delta K}$). Images were generated by MOE program suite (Ver. 2016.08, Chemical Computing Group Inc., https://www.chemcomp.com).[18] Quartet (WT) or triplet (ΔK) lysine residues are represented as magenta sticks. Orange and green spheres are hydrophobic residues on two different peptide chains that assemble into a dimeric coiled-coil structure.

Supplementary Figure 11. Full western blot images for Supplementary Fig. 6. Full-length images corresponding to cropped blots of **(A)** smooth muscle myosin heavy chain isoform 1 (SM1) (Lanes 2 and 3 = WT. Lanes 6 and 7 = *Myh11*$^{\Delta K/\Delta K}$) and **(B)** smooth muscle myosin heavy chain isoform 2 (SM2) shown in Supplementary Fig. 6. (Lanes 2 and 3 = WT. Lanes 6 and 7= *Myh11*$^{\Delta K/\Delta K}$). The red boxes denote the regions used in Fig. 6.

Supplementary Figure 12. Full western blot images for Supplementary Fig. 6. Full-length images corresponding to cropped blots of **(A)** α-actin (Lanes 1 and 2 = WT. Lanes 9 and 10 = *Myh11*$^{\Delta K/\Delta K}$) and **(B)** calponin shown in Supplementary Fig. 6. (Lanes 1 and 2 = WT. Lanes 9 and 10 = *Myh11*$^{\Delta K/\Delta K}$). The red boxes denote the regions used in Fig. 6.

Supplementary Figure 13. Full western blot images for Supplementary Fig. 6. Full-length images corresponding to cropped blots of **(A)** phosphorylated focal adhesion kinase (pFAK) (Lanes 1 and 2 = WT. Lanes 5 and 6 = *Myh11*$^{\Delta K/\Delta K}$) and **(B)** focal adhesion kinase (FAK) shown in Supplementary Fig. 6. (Lanes 2 and 3 = WT. Lanes 8 and 9 = *Myh11*$^{\Delta K/\Delta K}$).

The red boxes denote the regions used in Fig. 6.

Supplementary Figure 14. Full western blot image for Supplementary Fig. 6. Full-length image corresponding to cropped blots of glyceraldehyde 3-phosphate dehydrogenase (GAPDH) shown in Supplementary Fig. 6. (Lanes 1 and 2 = WT. Lanes 9 and 10 = *Myh11*$^{\Delta K/\Delta K}$). The red boxes denote the regions used in Fig. 6.

Supplementary Movie

Beating cells having differentiated in a WT embryoid body. The movie shows beating cells that have differentiated in a WT embryoid body. The hanging drop method was used to generate EBs.

Supplementary References

1 Schindelin, J. *et al.* Fiji: an open-source platform for biological-image analysis. *Nature Methods* **9**, 676-682, doi:10.1038/nmeth.2019 (2012).

2 Preibisch, S., Saalfeld, S. & Tomancak, P. Globally optimal stitching of tiled 3D microscopic image acquisitions. *Bioinformatics* **25**, 1463-1465, doi:10.1093/bioinformatics/btp184 (2009).

3 Kume, S. *et al.* in *Semantic Technology Lecture Notes in Computer Science* Ch. Chapter 19, 277-285 (2017).

4 Lu, H. *et al.* Subcutaneous Angiotensin II Infusion using Osmotic Pumps Induces Aortic Aneurysms in Mice. *J Vis Exp*, doi:10.3791/53191 (2015).

5 Hasegawa, A., Daub, C., Carninci, P., Hayashizaki, Y. & Lassmann, T. MOIRAI: a compact workflow system for CAGE analysis. *BMC Bioinformatics* **15**, 144, doi:10.1186/1471-2105-15-144 (2014).

6 Mi, H., Muruganujan, A., Ebert, D., Huang, X. & Thomas, P. D. PANTHER version 14: more genomes, a new PANTHER GO-slim and improvements in enrichment analysis tools. *Nucleic Acids Res* **47**, D419-D426, doi:10.1093/nar/gky1038 (2019).

7 Carbon, S. *et al.* AmiGO: online access to ontology and annotation data. *Bioinformatics* **25**, 288-289, doi:10.1093/bioinformatics/btn615 (2009).

8 Szklarczyk, D. *et al.* STRING v11: protein-protein association networks with increased coverage, supporting functional discovery in genome-wide experimental datasets. *Nucleic Acids Res* **47**, D607-D613, doi:10.1093/nar/gky1131 (2019).

9 Szklarczyk, D. *et al.* The STRING database in 2017: quality-controlled protein-protein association networks, made broadly accessible. *Nucleic Acids Res* **45**, D362-D368, doi:10.1093/nar/gkw937 (2017).

10 Franceschini, A. *et al.* STRING v9.1: protein-protein interaction networks, with increased coverage and integration. *Nucleic Acids Res* **41**, D808-815, doi:10.1093/nar/gks1094 (2013).

11 Franceschini, A., Lin, J., von Mering, C. & Jensen, L. J. SVD-phy: improved prediction of protein functional associations through singular value decomposition of phylogenetic profiles. *Bioinformatics* **32**, 1085-1087, doi:10.1093/bioinformatics/btv696 (2016).

12 Jensen, L. J. *et al.* STRING 8--a global view on proteins and their functional interactions in 630 organisms. *Nucleic Acids Res* **37**, D412-416,

doi:10.1093/nar/gkn760 (2009).

13 von Mering, C. *et al.* Genome evolution reveals biochemical networks and functional modules. *Proc Natl Acad Sci U S A* **100**, 15428-15433, doi:10.1073/pnas.2136809100 (2003).

14 von Mering, C. *et al.* STRING 7--recent developments in the integration and prediction of protein interactions. *Nucleic Acids Res* **35**, D358-362, doi:10.1093/nar/gkl825 (2007).

15 Snel, B., Lehmann, G., Bork, P. & Huynen, M. A. STRING: a web-server to retrieve and display the repeatedly occurring neighbourhood of a gene. *Nucleic Acids Res* **28**, 3442-3444, doi:10.1093/nar/28.18.3442 (2000).

16 Szklarczyk, D. *et al.* STRING v10: protein-protein interaction networks, integrated over the tree of life. *Nucleic Acids Res* **43**, D447-452, doi:10.1093/nar/gku1003 (2015).

17 Takahashi, K. & Yamanaka, S. Induction of pluripotent stem cells from mouse embryonic and adult fibroblast cultures by defined factors. *Cell* **126**, 663-676, doi:10.1016/j.cell.2006.07.024 (2006).

18 Chemical Computing Group ULC. Molecular Operating Environment (MOE), 2016.08. 010 Sherbooke St. West, Suite #910, Montreal, QC, Canada, H3A 2R7, https://www.chemcomp.com/.

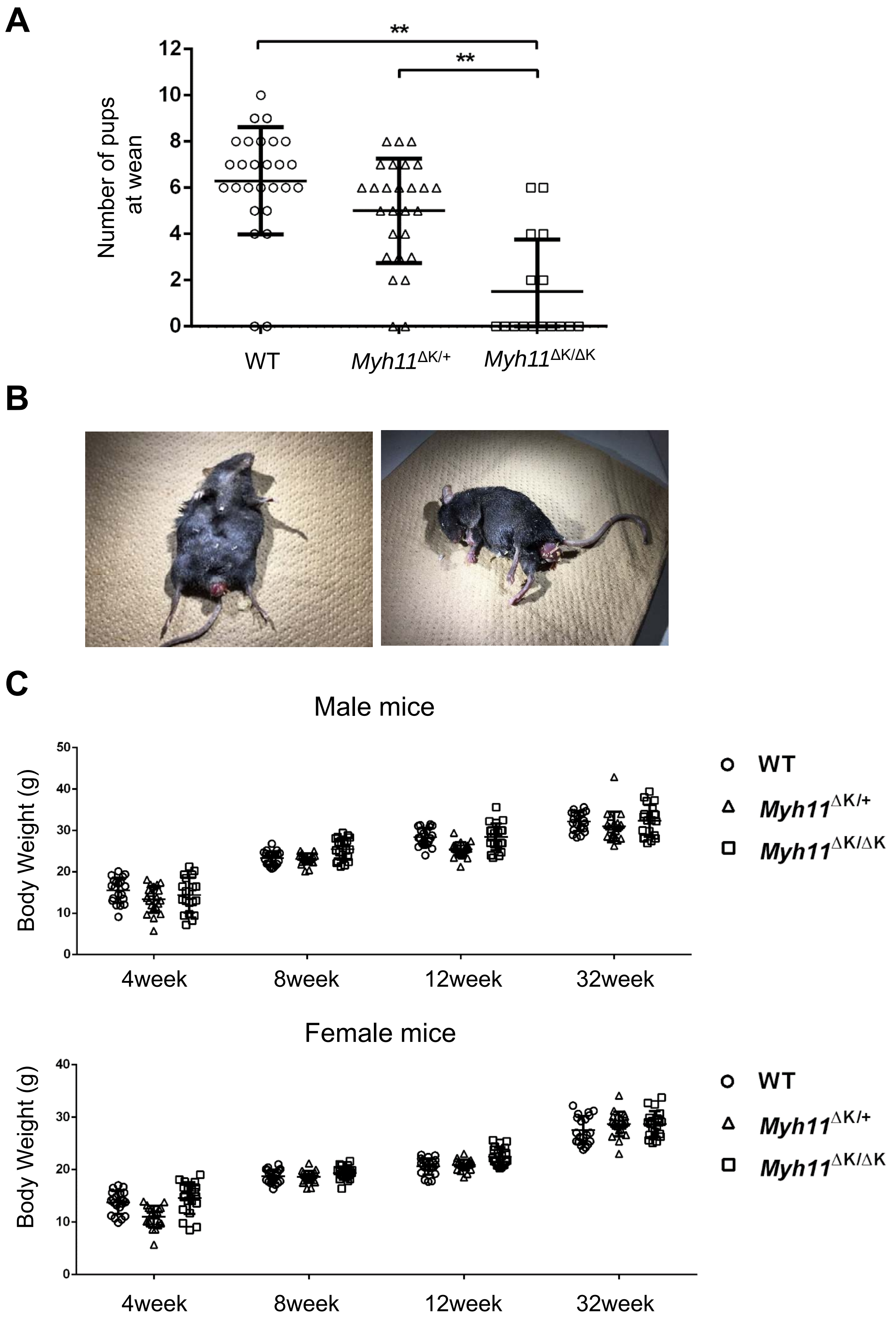

Supplementary figure 1

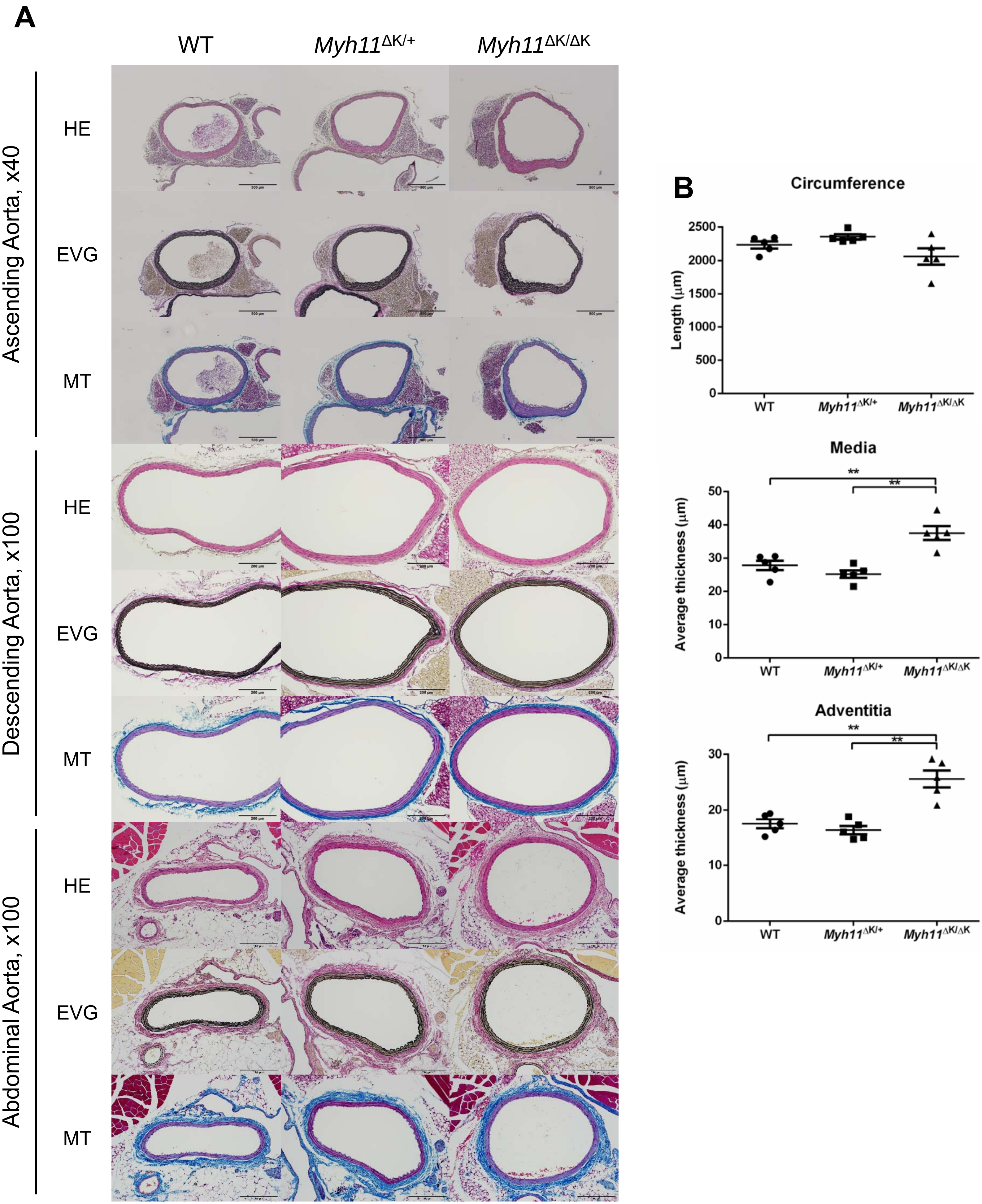

Supplementary figure 2

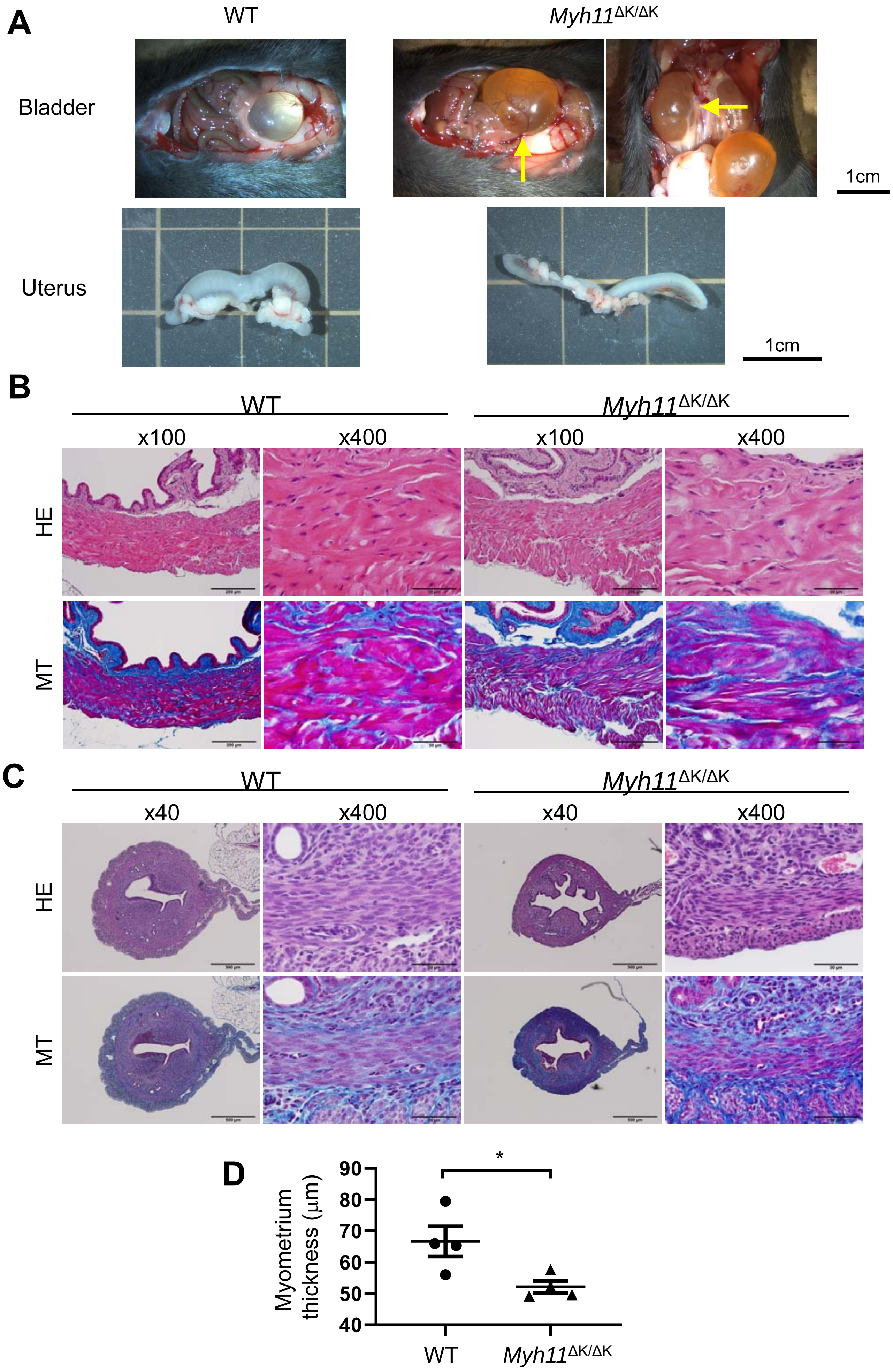

Supplementary figure 3

A

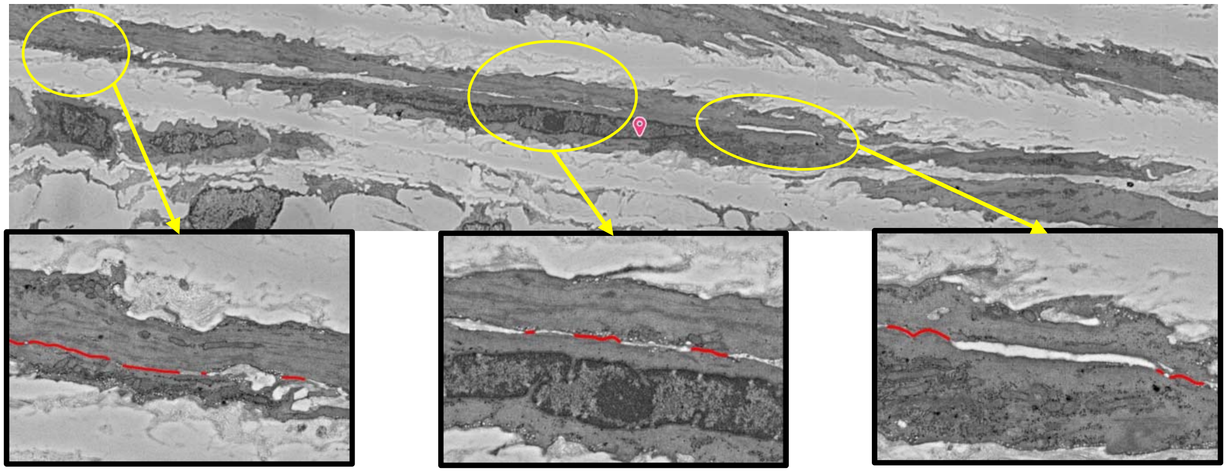

B

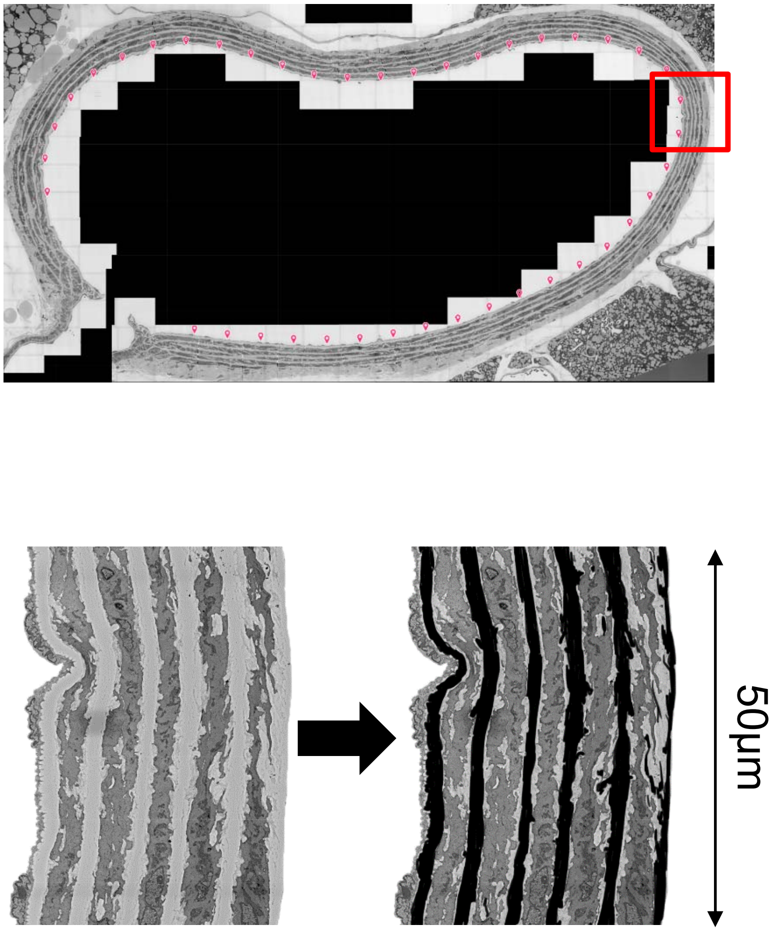

Supplementary figure 4

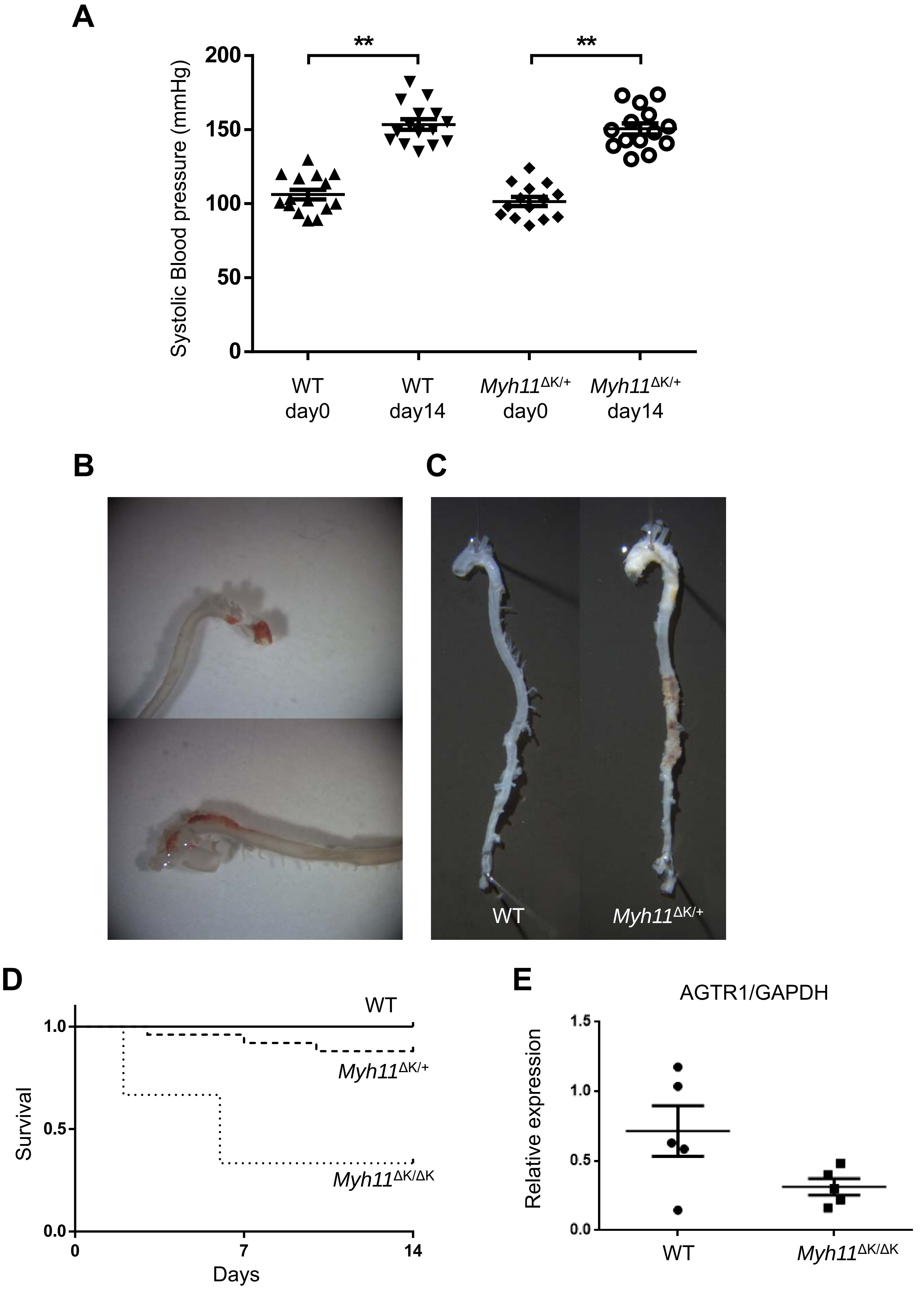

Supplementary figure 5

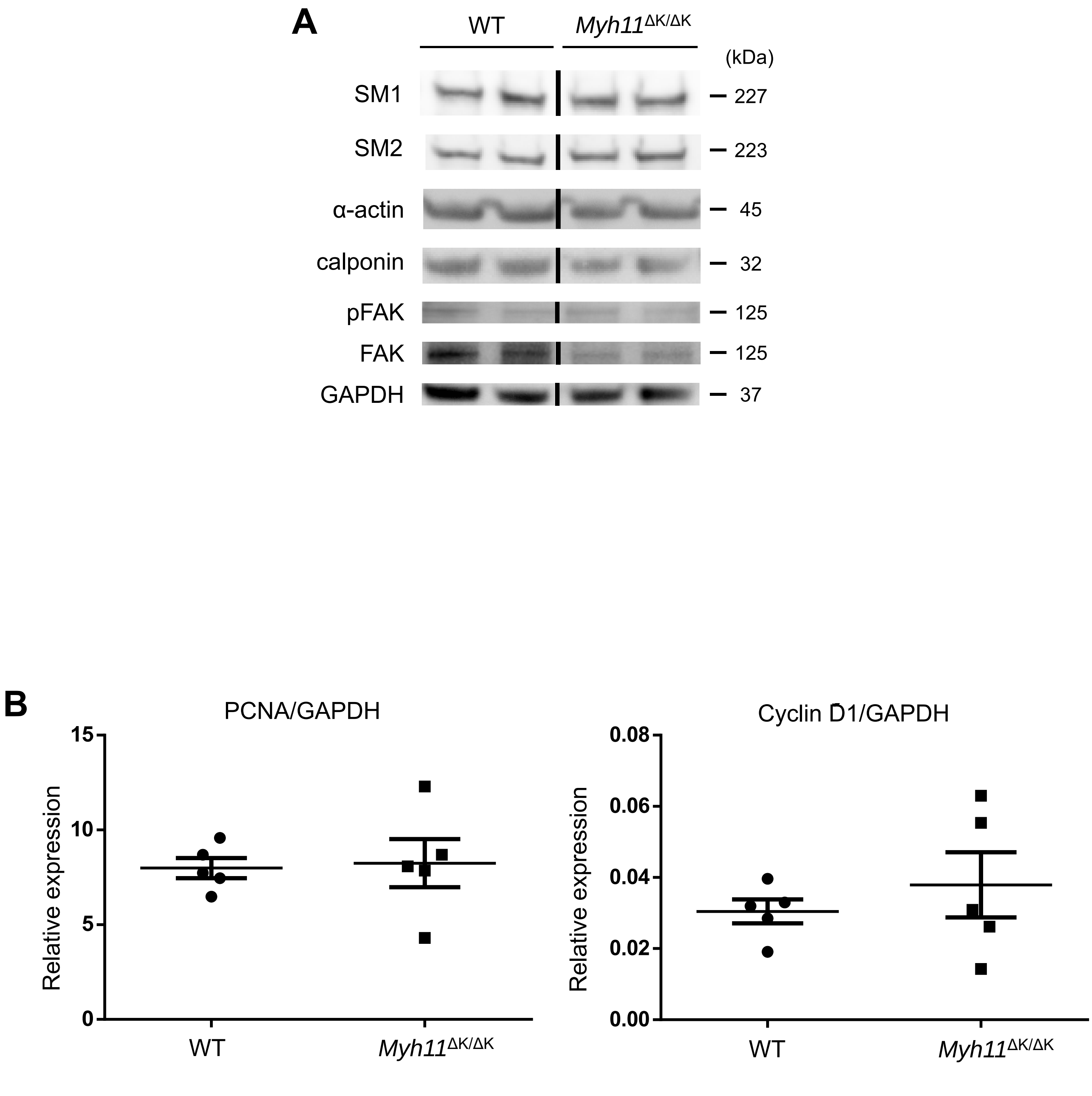

Supplementary figure 6

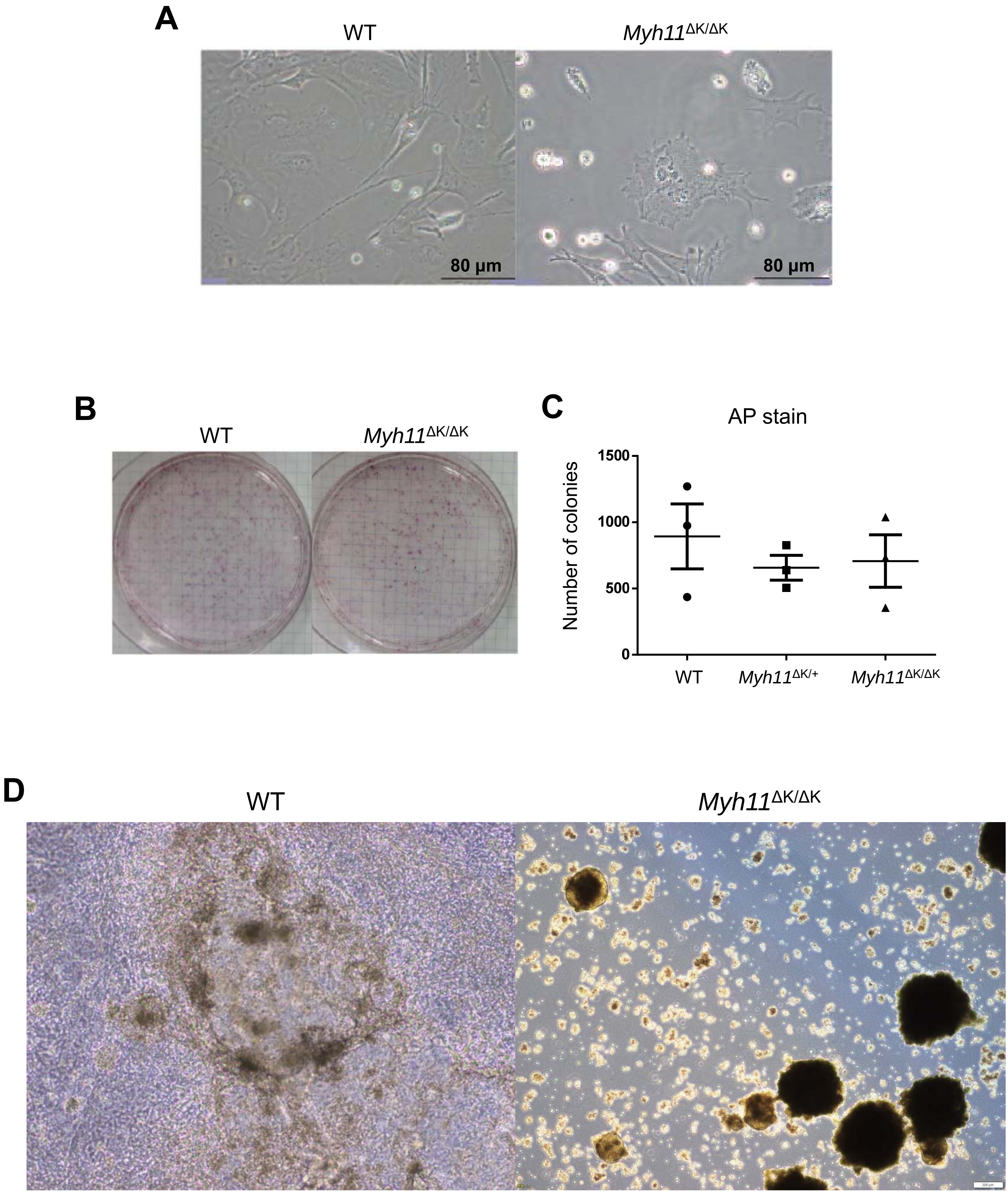

Supplementary figure 7

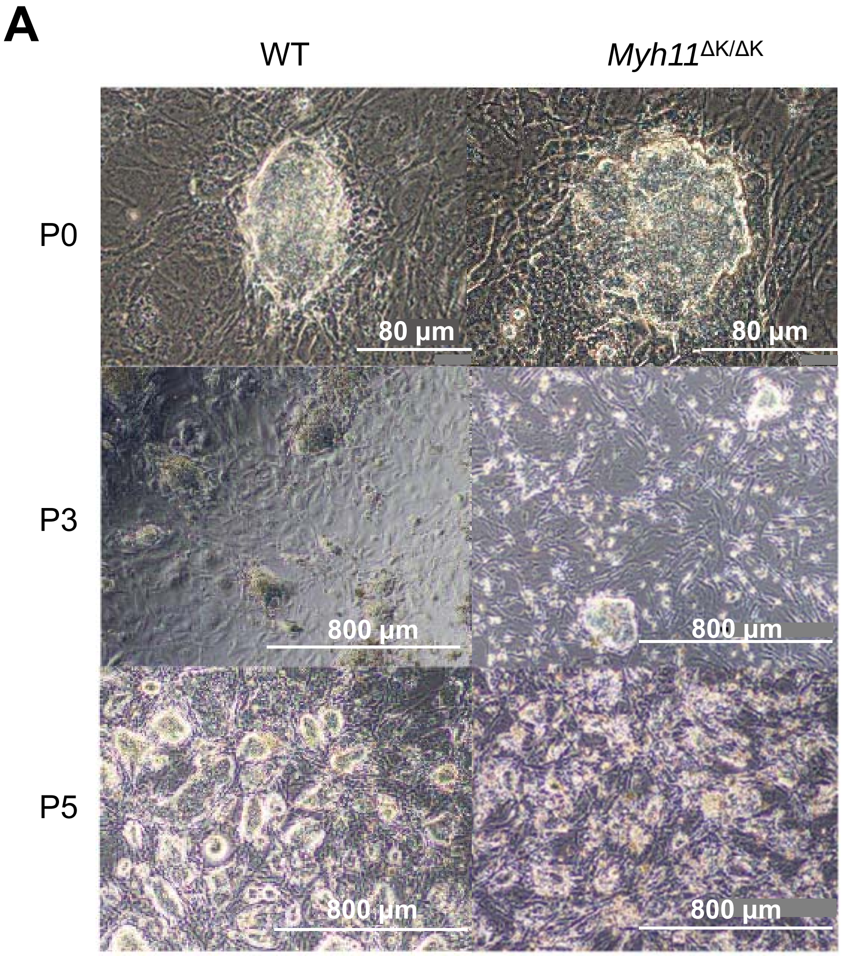

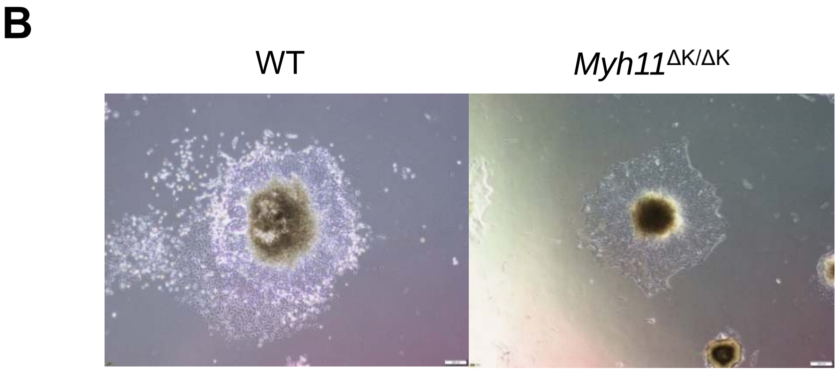

Supplementary figure 8

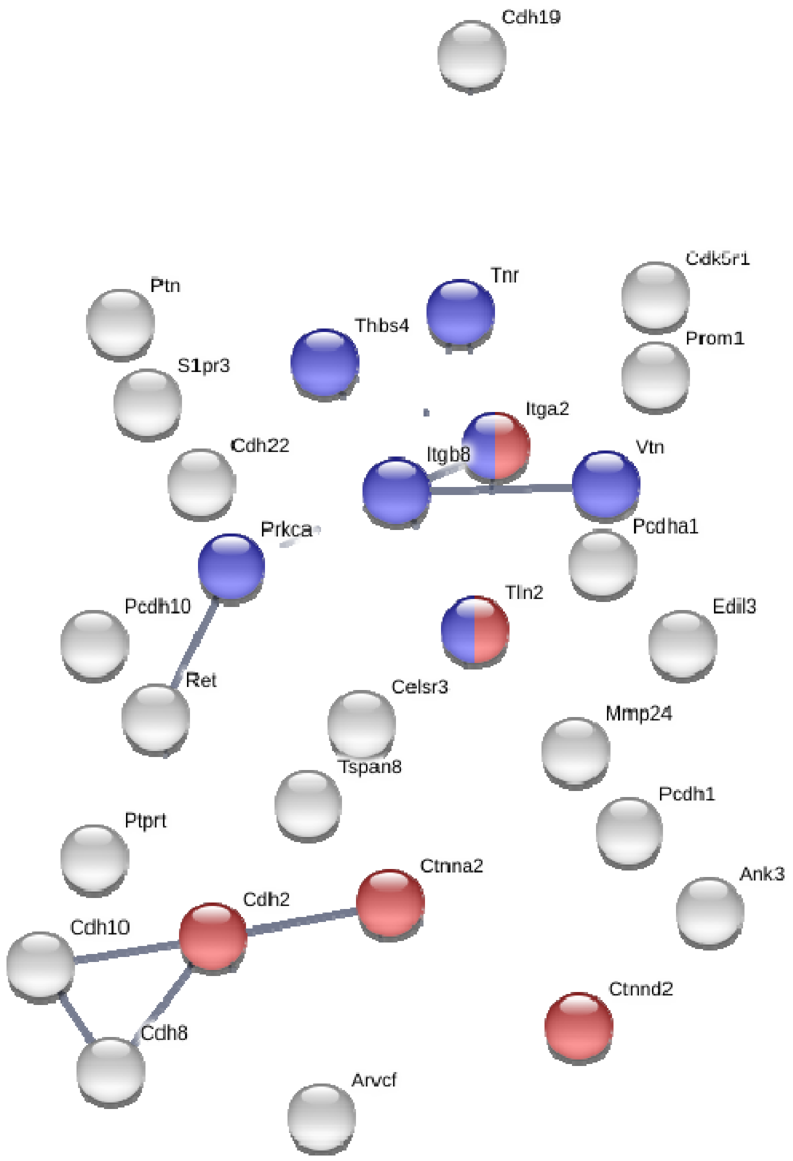

Supplementary figure 9

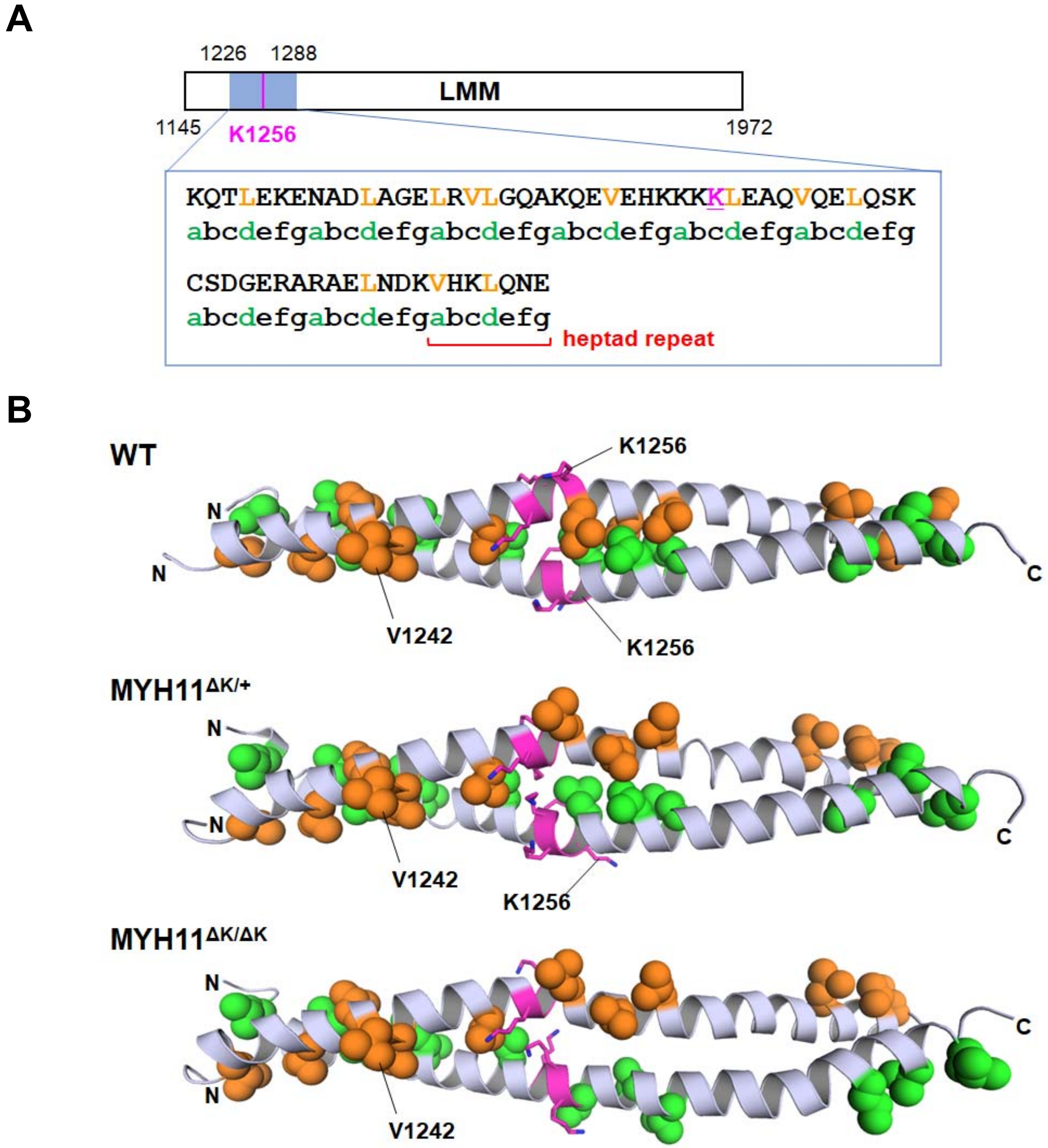

Supplementary figure 10

A

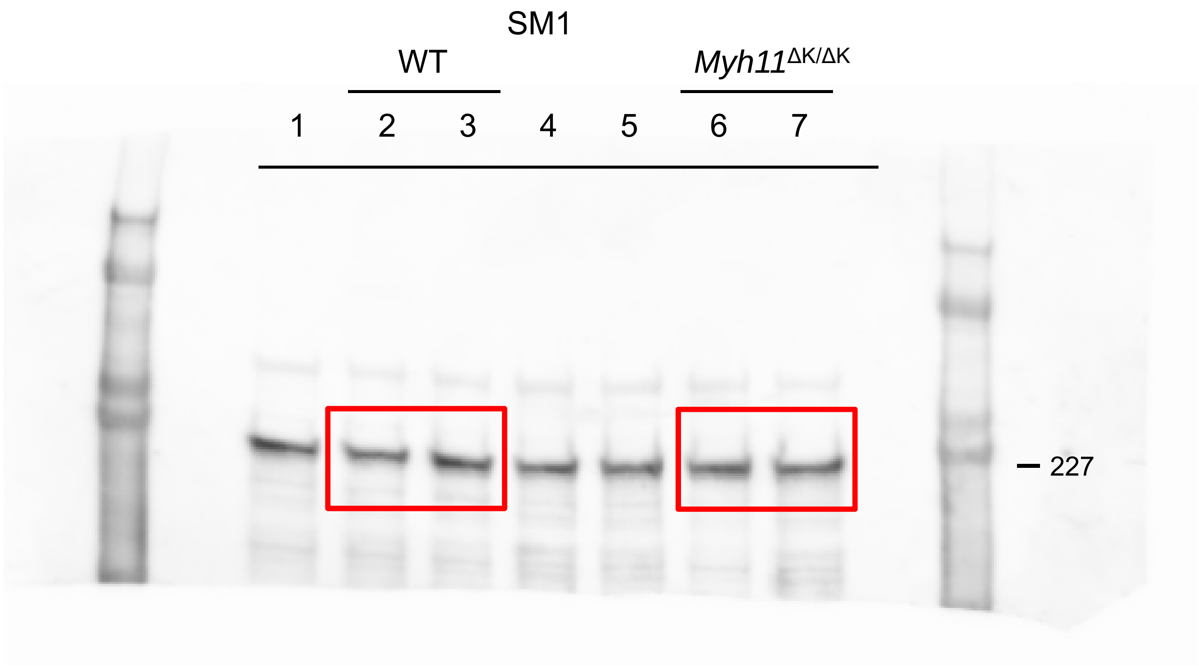

B

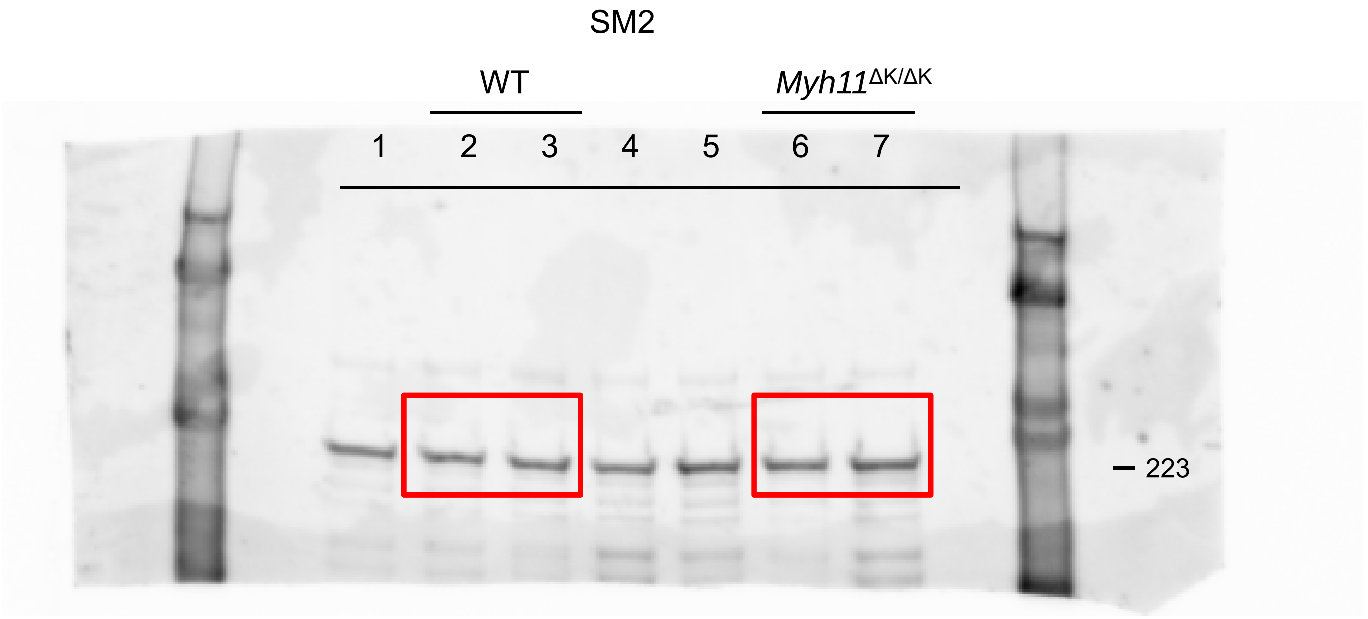

Supplementary figure 11

A

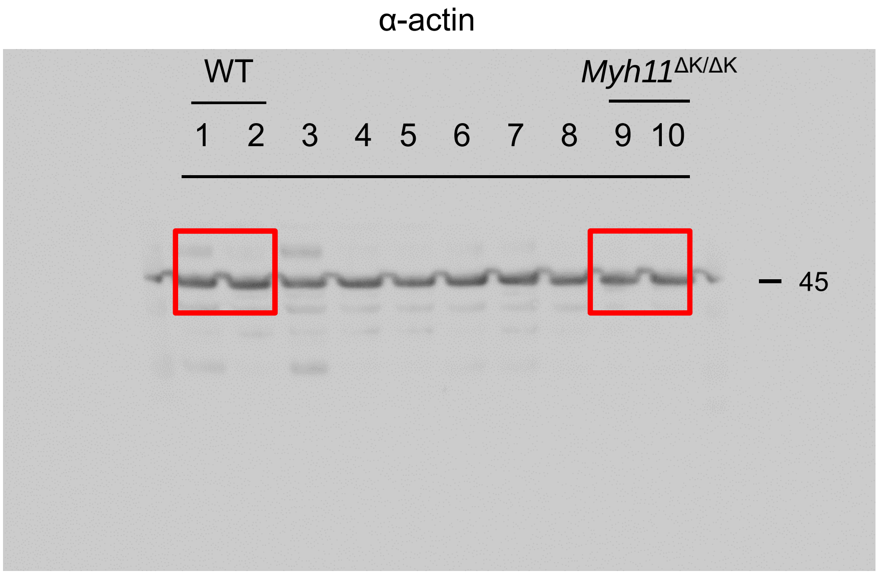

B

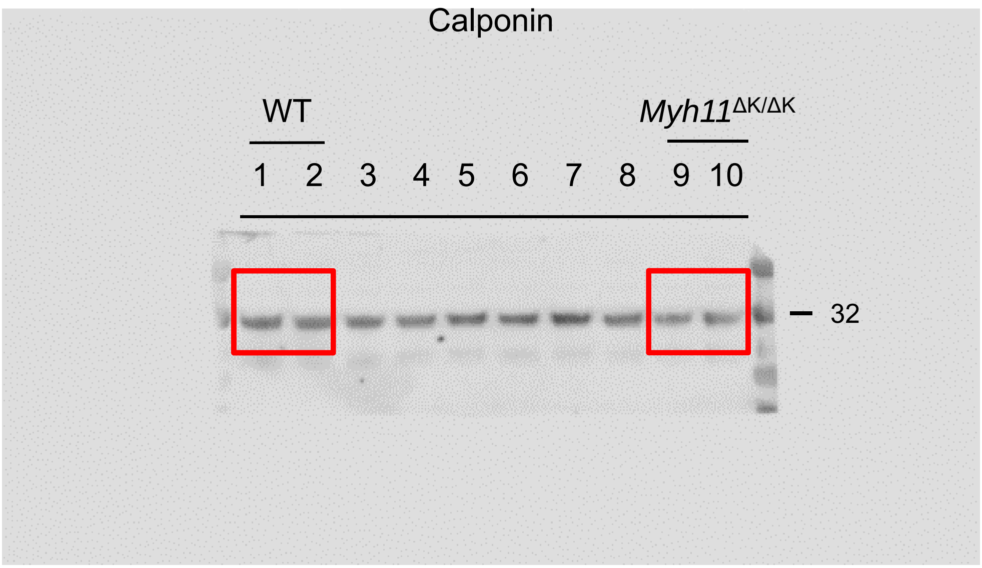

Supplementary figure 12

A

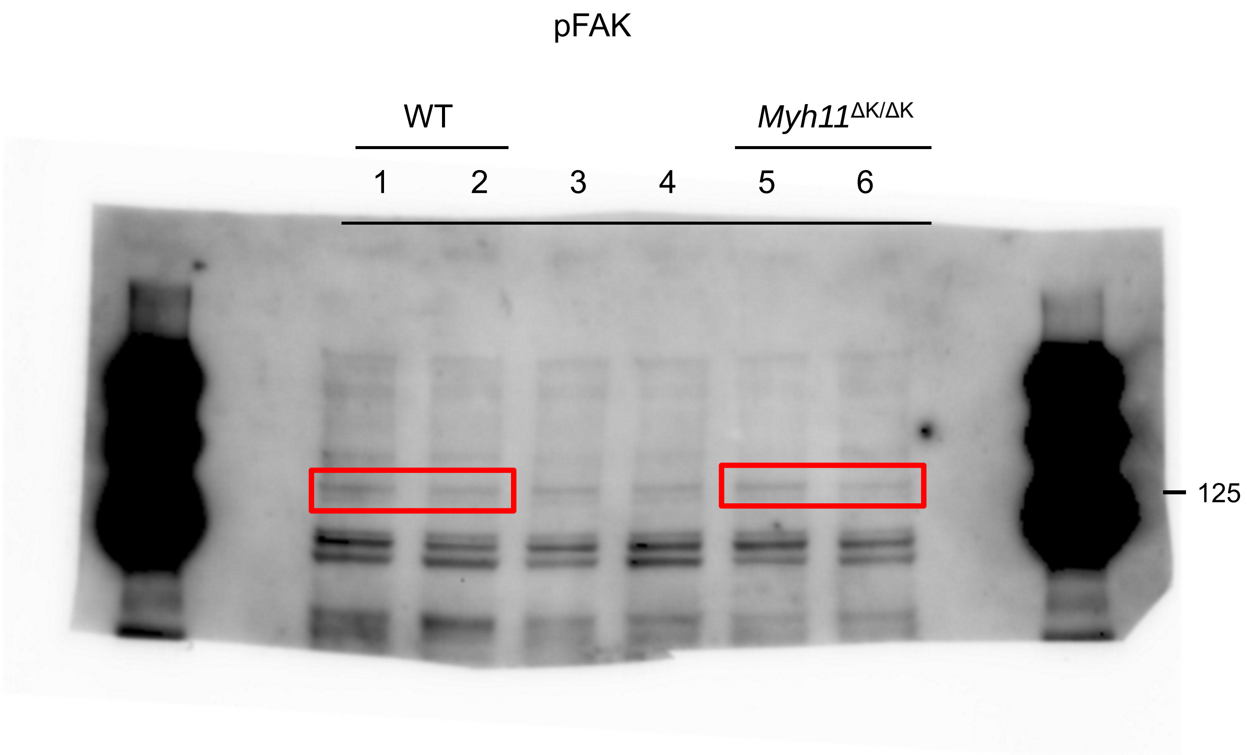

B

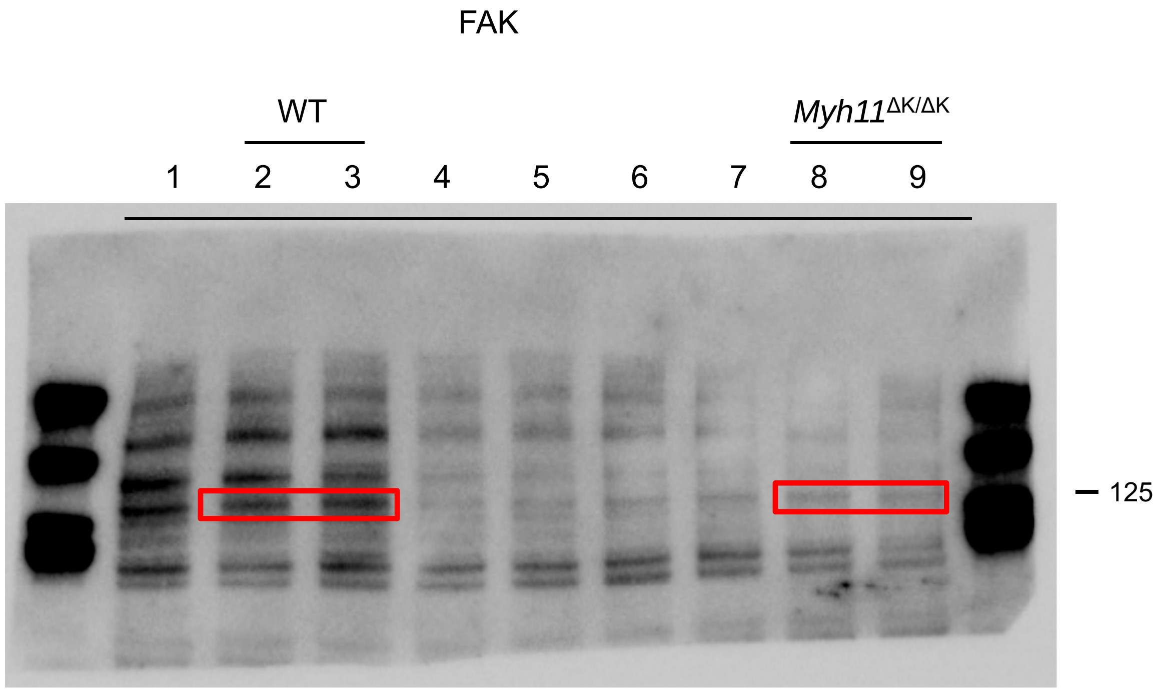

Supplementary figure 13

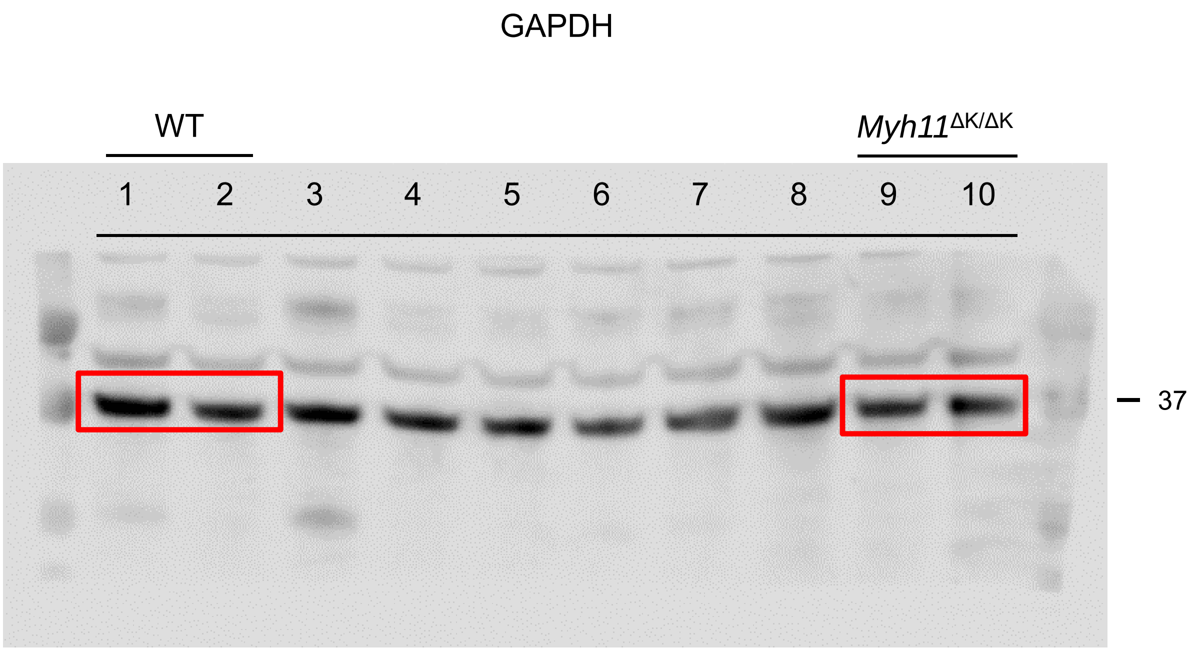

Supplementary figure 14
